# Amantadine: reappraisal of the timeless diamond—target updates and novel therapeutic potentials

**DOI:** 10.1007/s00702-021-02306-2

**Published:** 2021-02-23

**Authors:** Wojciech Danysz, Andrzej Dekundy, Astrid Scheschonka, Peter Riederer

**Affiliations:** 1 Merz Pharmaceuticals GmbH., Eckenheimer Landstraße 100, 60318 Frankfurt am Main, Germany; 2 Clinic and Policlinic for Psychiatry, Psychosomatics and Psychotherapy, University Hospital Würzburg, University of Würzburg, Margarete-Höppel-Platz 1, 97080 Würzburg, Germany; 3 Department Psychiatry, University of Southern Denmark Odense, Vinslows Vey 18, 5000 Odense, Denmark

## Abstract

The aim of the current review was to provide a new, in-depth insight into possible pharmacological targets of amantadine to pave the way to extending its therapeutic use to further indications beyond Parkinson’s disease symptoms and viral infections. Considering amantadine’s affinities in vitro and the expected concentration at targets at therapeutic doses in humans, the following primary targets seem to be most plausible: aromatic amino acids decarboxylase, glial-cell derived neurotrophic factor, sigma-1 receptors, phosphodiesterases, and nicotinic receptors. Further three targets could play a role to a lesser extent: NMDA receptors, 5-HT3 receptors, and potassium channels. Based on published clinical studies, traumatic brain injury, fatigue [e.g., in multiple sclerosis (MS)], and chorea in Huntington’s disease should be regarded potential, encouraging indications. Preclinical investigations suggest amantadine’s therapeutic potential in several further indications such as: depression, recovery after spinal cord injury, neuroprotection in MS, and cutaneous pain. Query in the database http://www.clinicaltrials.gov reveals research interest in several further indications: cancer, autism, cocaine abuse, MS, diabetes, attention deficit-hyperactivity disorder, obesity, and schizophrenia.

## Introduction

### Highlights and lowlights of drug development

Drug development and introduction of new treatments bear some similarity to sports. In the first half of twentieth century, Olympic long jump records were beaten, sometimes markedly, at nearly every Olympic Games. However, the Bob Beamon’s long jump Olympic record from 1968 (8.9 m) is still standing (Wikipedia 2020). Similarly, in the twentieth century, numerous new drugs were introduced at a very fast pace often turned out to be breakthrough therapies. However, nowadays, this is no longer the case because of the following reasons:

1. There are plenty of drugs on the market, many of them already generics with good efficacy.
2. In turn, it is more and more difficult to develop treatment that is clearly superior to existing generics to justify high pricing allowing return of investment.
3. It seems that novel treatments for neurological and psychiatric disorders must be multifactorial, and it is not possible to develop such substances by high-throughput screening.
4. On top of that, regulatory requirements for approval of a new drug were significantly raised which dramatically increased the costs and development time especially regarding limited duration of patent validity.

This clearly leads to the necessity to look back at existing drugs and explore their alternative, potential indications. This process is called repurposing and amantadine may serve as an interesting example thereof. In fact, amantadine was first introduced for influenza and later due to clinical observations found to be beneficial for the treatment of Parkinson’s disease. The present review is focusing on discussion of further plausible indications of this compound in relation to reappraisal of its mechanism of action.

### Historical introduction to amantadine

**Fig. 1:**
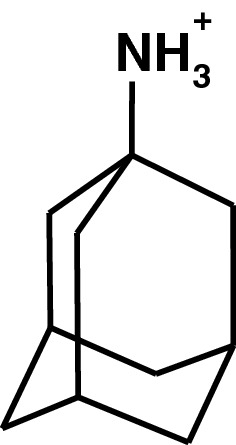
Chemical structure of amantadine (1-aminoadamantane)

Amantadine (Fig. 1) was initially developed in early 60s and registered for anti-influenza A2 activity in 1966 (Gerzon et al. 1963; Maj et al. 1974). Few years later, a woman suffering from Parkinson’s disease observed radical improvement of symptoms after taking amantadine taken for ani-viral purposes and reported it to Schwab et al. Following that, the authors performed a clinical study on 163 patients with a positive outcome (Schwab et al. 1969). Following that, he performed a clinical study on 163 patients with a positive outcome. A few years later, amantadine was registered for use in Parkinson’s disease. In the next 5 decades, there were many clinical studies of amantadine in various indications which are discussed later in this review. Selected discoveries related to increasing our knowledge on amantadine or extending therapeutic use are listed in Table 1.

**Table 1:** Chronology of major discoveries/findings related to amantadine

Year	Discovery description	References
1963	First description of amantadine as medication—antiviral activity	Gerzon et al. (1963)
1966	Amantadine (Symmetrel) by E. I. du Pont de Nemours & Company and approved by FDA in the USA for prevention of influenza A2	Hubsher et al. (2012) and Maugh (1979)
1969	First description of antiparkinsonian activity based on case observation (1968) by woman taking amantadine for viral infection	Schwab et al. (1969)
1970	Registered by Merz Pharmaceuticals as PK-Merz (amantadine sulfate) for the treatment of Parkinson’s disease, vigilance and neuralgia in herpes zoster	Zimmermann (1997)
1971	Effect in tardive dyskinesia	Crane (1971)
1971	Effect on chorea in Huntington's disease	Scotti and Spinnler (1971)
1073	Effect in akinetic crisis and akinetic end stages of patients with Parkinson’s disease	Danielczyk (1973)
1973	Amantadine hydrochloride (Symmetrel) registered as treatment for Parkinson’s disease	Hubsher et al. (2012) and Maugh (1979)
1981	Anti-tremor activity	Manyam (1981)
1989	First report of NMDA receptor binding by amantadine	Kornhuber et al. (1989)
1989	Utility in traumatic brain injury (TBI)	Gualtieri et al. (1989)
1991	First demonstration of NMDA receptor antagonism by amantadine	Kornhuber et al. (1991)
1993	Binding to sigma receptors	Kornhuber et al. (1993)
1997	Effect in l-DOPA-induced dyskinesia	Rajput et al. (1997)
2020	Approval of Gocovri (ADS-1502, Adamas Pharma) for treatment of DOPA-induced dyskinesia	Adamas (2020)

In recent years, a lot of attention has been devoted to efficacy in traumatic brain injury (TBI) of different origins (Butterworth 2020a; Gualtieri et al. 1989). As follow on, cellular protection has been proposed in various insults or neurodegenerative conditions including Parkinson’s disease, stroke, and infectious disease (Brison et al. 2014; Butterworth 2020c; Khasanova et al. 2009; Quarato et al. 2014; Rejdak and Grieb 2020; Uitti et al. 1993). Independently, very recently, putative activity of amantadine against SARS-CoV-2 (COVID-19) has been described and widely discussed (Butterworth 2020c; Rejdak and Grieb 2020).

It should be noted that there are two amantadine salts on the market: amantadine hydrochloride originally introduced by Dupont as Symmetrel and amantadine sulfate introduced by Merz Pharmaceuticals as PK-Merz (Table 1, Fig. 1). It is noteworthy that after oral treatment, the increase in plasma levels after amantadine sulfate (PK-Merz) is more gradual and lasts longer due to slower absorption which is likely the result of lower solubility (Danielczyk 1995).

### Aim of the review

The first goal of the present review was to analyze possible molecular targets of amantadine considering actual therapeutic concentrations and to accordingly reappraise its mechanism of action. The second goal was to collect data supporting clinical and preclinical effects in indications beyond viral infections and Parkinsons disease and to discuss them in the light of the updated view on the mechanism of action. The ultimate aim is to propose the most promising indications to encourage studies leading to the expansion of indications.

Readers interested in antiviral or antiparkinsonian/antidyskinetic activity should refer to one of the previously published reviews (Aranda-Abreu et al. 2020b; Bailey and Stone 1975; Butterworth 2020b; Crosby et al. 2003; Danielczyk 1995; Danysz et al. 1997; Hubsher et al. 2012; Kornhuber et al. 1994; Schwab et al. 1972; Smieszek et al. 2020; Stanicova et al. 2001; Tipton and Wszolek 2020).

## Amantadine therapeutic concentrations (animal and human data)

See Tables 2 and 3.

**Table 2:** Plasma/serum/tissue concentrations of amantadine in animals

Species	Dose (mg/kg), route	Use	Body fluids, µM	Brain—CSF/ECF, µM	Brain homog, µM	References
Rat	25, p.o. 50, p.o. 100, p.o.	Antiparkinsonian-like activity	4.5 (s) 10.5 (s) 21.0 (s)		– 90.0 –	Danysz et al. (1994b)
	15, i.p. 45, i.p. 90, i.p.	Pharmacokinetics in relation to antidyskinetic effects	14.8 (p) 37.2 (p) 132.6 (p)			Brigham et al. (2018)
	100 (s.c.)	PK study, infusion, dose per day	ca. 8 (s)	ca. 4.6/4.0	ca. 70	Hesselink et al. (1999)
	23, i.p. 46, i.p. 92, i.p.	Antiparkinsonian-like activity, pharmacokinetics, microdialysis		7.7 11.9 23.1		Kornhuber et al. (1995) and Quack et al. (1995)
	5, i.p. 15, i.p. 45, i.p.	TBI, repetitive treatment 3 × per day for 16 days after TBI	5.3 (s) 21.3 (s) 63.9 (s)			Wang et al. (2014)
	100, s.c.	Neuroprotection study, dose per day—infusion for 14 days	8.77 (s)		107.5	Wenk et al. (1996)
Mouse	25, p.o.	Pharmacokinetics	42.7 (b)			Bleidner et al. (1965)
	10, i.p. 30, i.p. 60, i.p.	Pharmacokinetics in relation to antidyskinetic effects	6.8 (p) 19.9 (p) 47 (p)			Brigham et al. (2018)
Macaques	1, i.p. 3, i.p. 10, i.p 30, i.p.	Pharmacokinetics in relation to antidyskinetic effects	0.86 p) 2.9 (p) 8.5 (p) 24.7 (p)			Brigham et al. (2018)

*s* serum, *p* plasma, *b* blood, *CSF* cerebrospinal fluid, *ECF* extracellular fluid, *homog.* homogenates

**Table 3:** Plasma/serum/tissue concentrations of amantadine in humans

Dose (mg)	Use	Body fluids, µM	Brain–CSF, µM	Brain homog, µM	References
200, p.o. > 8 days 300, p.o	Antiparkinsonian	5.0 (s) 13.5 (s)	4.0 9.5	159 281	Kornhuber et al. (1995)^a^
300, p.o. 3 weeks	Antidepressive	3.6–5.4 (p)			Rizzo et al. (1973)
200, i.v. 600, p.o. 1–24 weeks	Antiparkinsonian	3–5 (s) 8–11 (s)	1.3		Brenner et al. (1989)
200, p.o. 2 weeks	Antagonism of drug-induced Parkinsonism	2.1–4.5 (p)			Pacifici et al. (1976)
ca. 280–350, p.o	Pharmacokinetics	2.7–3.2 (b)			Bleidner et al. (1965)
274, p.o. ER	Pharmacokinetics extended release for 8 weeks in PD patients	ca. 7.5 (p)			Hauser et al. (2019)
129, p.o. ER 193, p.o. ER 259, p.o. ER 129, p.o. IR (BID)	Pharmacokinetics extended release vs. immediate release	1.74 (p) 2.45 (p) 3.35 (p) 2.15 (p)			deVries et al. (2019)
50–300, p.o.	78 Parkinsonian patients	5.3 (average p)			Nishikawa et al. (2009)
200, p.o. 300, p.o.	Side effects in comparison to rimantadine. Values after 9 doses	4.8 (p) 9.3 (p)			Hayden et al. (1983)
100, p.o.	Efficacy and PK in tardive dyskinesia	2.9 (p)			Greenblatt et al. (1977)
100, p.o. BID	Pharmacokinetics, interaction with oseltamivir, treatment for 5 days	3.39 (p)			Morrison et al. (2007)
350, p.o. (average)	Antidyskinetic activity	4–23 µM			Verhagen Metman et al. (1998)
50, p.o. 200, p.o. 300, p.o. 15 days steady state	Influenza A	0.59 (p) 1.62 (p) 3.06 (p)			Aoki and Sitar (1988) and Aoki et al. (1979)

*s* serum, *p* plasma, *b* blood, *BID* twice daily, *CSF* cerebrospinal fluid, *IS* interstitial fluid, *homog.* homogenates

^a^The values given for brain homogenates are not derived from the same group of patients as serum and CSF levels

^b^CSF levels specified as 1.3 times higher than serum

## Plausible mechanism of therapeutic action of amantadine

The mechanism of action (MoA) of amantadine has to be analyzed in relation to its concentrations reached at a given target in humans (Table 3) following therapeutic doses and its affinity at the target (Table 4). With this information, one can judge the plausibility of the targets responsible for the mechanism of action.

**Table 4:**
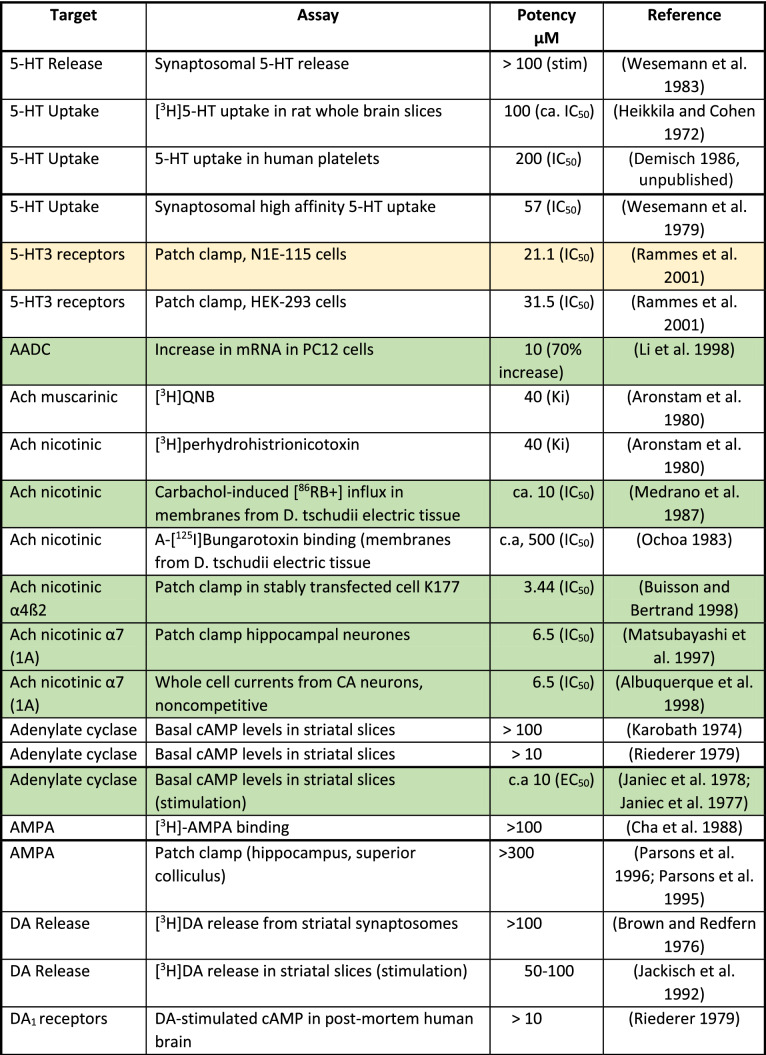

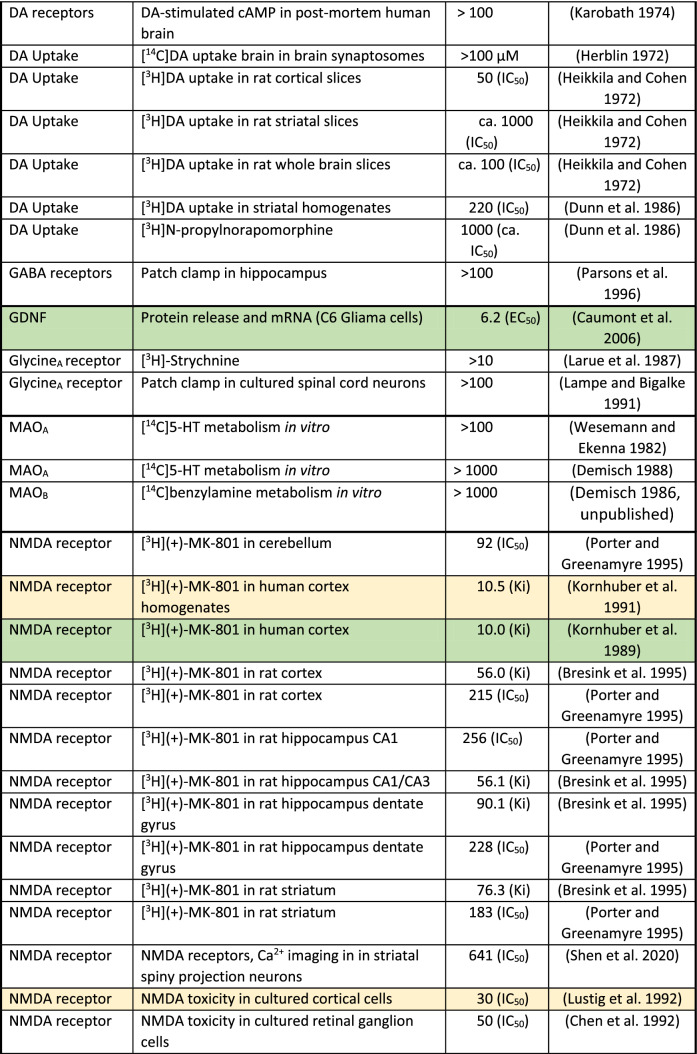

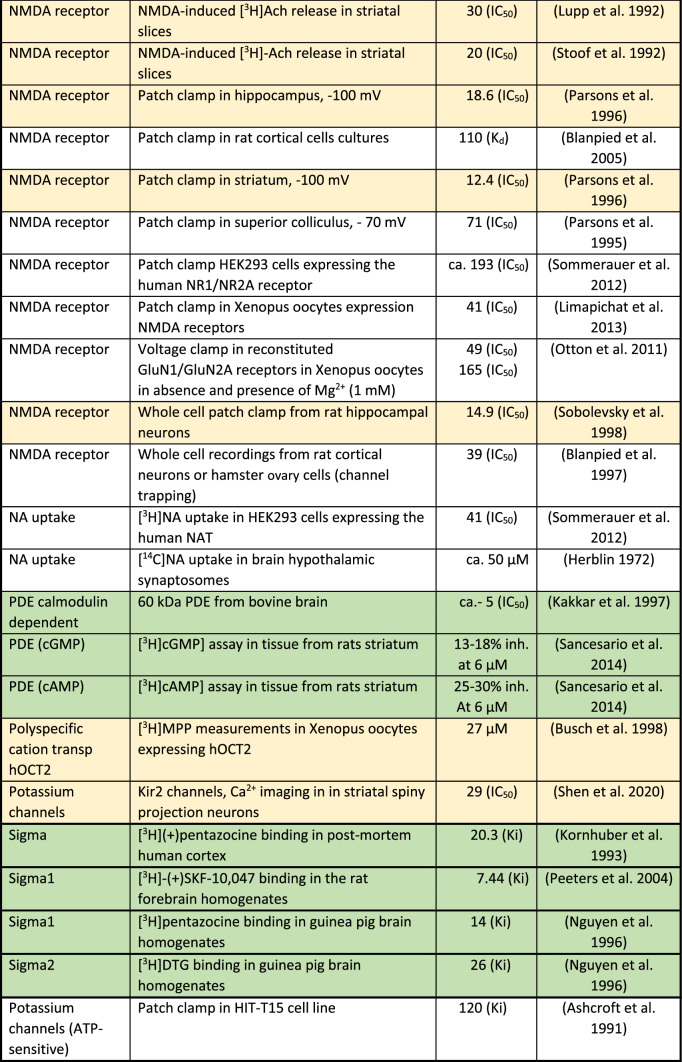
Compilation of in vitro actions of amantadine

ca.—the approximate concentration that produces 50% inhibition or stimulation

Extracellular targets with affinity up to 10 µM and intracellular up to 200 µM were considered as certain and are indicated in green. In yellow, intracellular targets with 10–30 µM affinity and intracellular between 200 and 600 µM are presented. All other targets remain white

Cerebrospinal fluid (CSF) levels of amantadine in the human brain are slightly (24%) lower than plasma levels (Kornhuber et al. 1995). Extracellular concentration has been assessed in animals using the microdialysis technique with in vivo recovery leading to values ranging from 2.2 to 6.4 µM (depending on recovery mode). In experimental study in rats, serum level of 8.7 µM was associated with CSF level of 7.5 µM (Hesselink et al. 1999).

However, intracellular concentrations are 10 or 20 times higher than plasma levels in animal and human studies, respectively, due to lysosomal trapping (Danysz et al. 1994b; Hesselink et al. 1999; Kornhuber et al. 1995). For lipophilic amines with Log*P* > 1 and ionizable amino group (p*K*A > 6), a strong concentration accumulation gradient is created as follows: lysosomes > cytosol > blood (Daniel et al. 2001; Stark et al. 2020). Amantadine fulfills these criteria with Log*P* of 2.44 and p*K*A of 10.45. Lysosomes have pH of 4–5, cytosol 7.0–7.2, and blood 7.4. Amantadine molecule in not-ionized state diffuses to lysosomes. It is then protonated, i.e., charged and not able to diffuse back to cytoplasm and later to circulation. In turn, a steady-state gradient is created leading to significant intracellular accumulation of amantadine. As a consequence, amantadine plasma concentrations which are below its in vitro affinity for given targets may still affect this target if it is intracellular, e.g., enzymes or receptors on endoplasmic reticulum. In Table 4, we took into consideration this aspect. In green, we marked up the targets with affinity within therapeutic concentration, i.e., which are affected at clinically used doses. In yellow, there are targets with affinity up to 3 times lower which could potentially play a supportive role. All other targets are left white. We set criteria for extracellular targets at 10 µM and below and for intracellular targets at 200 µM and below.

It should be also kept in mind that amantadine is an agent with multiple targets. Therefore, it may not be possible to explain given therapeutic efficacy by a single target but rather by a combination of actions.

In early preclinical studies, it was reported that amantadine causes excitation in rats and mice and—at higher dose—enhances motor behavior (Vernier et al. 1969) and that it potentiates the L-DOPA-induced effects in mice (Svensson and Stromberg 1970). As the effect of amantadine on motor behavior could not be abolished by pre-treatment with reserpine, but was antagonized by pre-treatment with ɑ-methyl-*p*-tyrosine, MoA at the catecholaminergic systems was suggested (Offermeier and Dreyer 1971). This action was also claimed to be responsible for a beneficial effect in Parkinson’s disease (ibid). In addition, it was suggested that amantadine increases the turnover of tyrosine to l-DOPA and enhances the synthesis of dopamine (Scatton et al. 1970). This was further supported by later findings showing changes in aromatic amino acids decarboxylase (Table 4). In rats and mice, amantadine even in low dose potentiates stereotypic behavior induced by amphetamine (Simon and Boissier 1970). In turn, amantadine was initially perceived as dopaminomimetic agent (Grelak et al. 1970; Herblin 1972; von Voigtlander and Moore 1973). However, studies of urine, CSF, and human post-mortem tissue of patients with Parkinson’s disease have not supported amantadine MoA via catecholaminergic or serotonergic systems (Jones et al. 1972; Parkes 1974; Rinne et al. 1972). Later, NMDA receptor antagonism was discovered (Kornhuber et al. 1989) and dominated in the scientific literature. In our opinion, NMDA antagonism probably is not the predominant mode of action.

In Table 4, there is only one out of 25 publications assessing in vitro NMDA receptor activity in green zone (up to 10 µM) and there are 8 out of 26 in the yellow zone (10–30 µM). This puts into question NMDA receptors as major target for therapeutic activity of amantadine. On top of that, it should be stressed that only a few studies were performed in the presence of physiological (1 mM) concentrations of Mg^2+^. In the study of Otton and colleagues (Otton et al. 2011), introduction of Mg^2+^ increased amantadine IC_50_ at NMDA receptors from 49 to 165 µM, i.e., over threefold. It should be stressed that plasma peak levels of amantadine reach maximally 10 µM and often are lower (Table 3). On the other hand, we do not know what levels of NMDA receptor inhibition are sufficient to produce an effect. Amantadine has been suggested to inhibit NMDA receptors by accelerating the channel closing, in turn leading to stabilization of the channel in the closed state (Blanpied et al. 2005).

There is ample evidence, indicating that amantadine profile is different from pure NMDA receptor antagonist.

1. Amantadine at 10 µM increased glutathione levels in astrocytes, while selective NMDA antagonist (+)(5S,10R)-(+)-5-methyl-10,11-dihydro-5*H*-dibenzo[a,d]cyclohepten-5,10-imine hydrogen maleate ((+)MK-801) was not effective even at 1 µM concentrations, i.e., over 500 times exceeding its affinity (Nakano et al. 2019).
2. In vivo, in microdialysis study in rats, the stimulatory effects of amantadine (10 or 40 mg/kg) on l-glutamate release were different from those of MK-801 in the globus pallidus, entorhinal cortex, and entopeduncular nucleus (Nakano et al. 2019).
3. There are clear differences in neuroprotective effects of amantadine, memantine, and (+)MK-801 between cerebellar, cortical, mesencephalic, and mesencephalic dopamine (DA) neurons, since amantadine in contrast to other two agents provided effect in the first two tissues only (Weller et al. 1993).
4. In rats, amantadine inhibited convulsions produced by NMDA with ED_50_ of 116 mg/kg, while memantine had ED_50_ of 4.6 mg (Parsons et al. 1995). In contrast, anti-parkinsonian-like effects of amantadine are seen starting from 25 mg/kg (Danysz et al. 1994b).
5. In rat Parkinson’s disease model, amantadine produced a different pattern of changes in STN than the selective NMDA receptor antagonist (+)MK-801 (Allers et al. 2005).
6. Amantadine antagonized the blood pressure responses evoked by electrical stimulation of the central and peripheral nerves possibly due to an effect on autonomic ganglia (Dhasmana 1975).
7. Amantadine in electrophysiological experiments has approx. 10 times stronger blocking affinity at cholinergic muscle plate than at NMDA receptors (Gmiro and Serdiuk 2000).
8. Amantadine at 15 or 30 mg/kg in rats decreased prolactin levels indicating enhancement of DA-ergic activity (Fayez et al. 1985; Siever 1981).
9. Amantadine produced a different pattern of locomotor activity changes in laboratory animals than selective NMDA receptor antagonists (Danysz et al. 1994a; Starr and Starr 1995).
10. Similar to apomorphine, amantadine given to rats at 20 mg/kg increased c-fos expression in the striatum 2 h later, while (+)MK-801 was devoid of this effect (Rappaport and Yells 1996). Interestingly, amantadine effect in the striatum was attenuated by the NMDA antagonist (+)MK-801 (Tomitaka et al. 1995).
11. Amantadine and memantine differently modulate dopaminergic transmission in the basal ganglia (Peeters et al. 2003) and amantadine-induced increase in DA in the striatum was antagonized by the selective NMDA receptor antagonist (+)MK-801 (Takahashi et al. 1996).

In conclusion, it is likely that NMDA receptor antagonism by amantadine possibly contributes dose-dependently to the therapeutic efficacy, but it is not its major mechanism. There are few other candidates affected by therapeutically relevant amantadine concentrations which are shortly discussed below and listed in Table 4:

1. *Aromatic amino acids decarboxylase (AADC)* AADC (which is an intracellular target) is responsible for the synthesis of dopamine (Fig. 3) and increases dopamine levels available for synaptic release. AADC expression (mRNA) is increased by amantadine (10 µM) by 70% in pheochromocytoma (PC12) cells (Li et al. 1998). Of course, it should be taken with caution, because effect on protein levels has not been shown so far in vitro and translational aspect of PC12 cells vs. human brain should be considered. In ex vivo animal study, amantadine at 40 mg/kg increased the activity of AADC threefold in the striatum and tenfold in the substantia nigra 1 h after injection (Fisher et al. 1998). This effect was not shared by selective NMDA antagonist (+)MK-801. In rats with 6-OHDA lesion to the dopaminergic system, amantadine at 30 mg/kg increased ex vivo AADC activity in the striatum as evidenced by L-DOPA conversion assay (Arai et al. 2003). This effect was not observed in the presence of benserazide which, according to the authors’ interpretation, argues against the role of AADC in the increase in striatal DA produced by amantadine. Further support comes from in vivo human study using 6-[^18^F]fluoro-l-DOPA (l-DOPA = 3,4-dihydroxy-l-phenylalanin), as exogenous substrate for AADC (Deep et al. 1999). Deep and colleagues found that amantadine given for 3 days at 100 mg increased the activity of AADC up to 27% in ventral striatum (Deep et al. 1999). The effect on AADC would obviously result in an increase in dopaminergic activity and could be clearly supportive in Parkinson’s disease and in other indications such as fatigue or recovery from TBI.
2. *Sigma 1 receptors* Amantadine is a very potent ligand of sigma-1 receptors (Fig. 3, Table 4) which has been first described by Kornhuber and colleagues (Kornhuber et al. 1993). These receptors are also located intracellularly, e.g., on membranes of endoplasmatic reticulum and control Ca^2+^ signaling (Monnet 2005). Sigma-1 receptors enhance tyrosine hydroxylase activity (Weiser et al. 1995), modulate NMDA stimulated DA release (Gonzalez-Alvear and Werling 1995), increase DA in vivo in the striatum (Gudelsky 1995), and decrease DA uptake (Thompson et al. 2001). All these effects may participate in the symptomatologic effects of amantadine in Parkinson’s disease. Sigma-1 receptors have been suggested as targets for neuroprotection in Parkinson’s disease (Francardo 2014; Mori et al. 2012; Rousseaux and Greene 2015). Sigma-1 agonists may be beneficial for: neuroprotection in general (Decoster et al. 1995; Maurice and Lockhart 1997), amyotrophic lateral sclerosis (ALS) (Mancuso et al. 2012), Alzheimer’s disease (Meunier et al. 2006), ischemia (Oneill et al. 1995), anxiety (Ji et al. 2017), and depression (Raupp-Barcaro et al. 2018; Skuza et al. 2014). Moreover, activation of sigma-1 receptors may enhance recovery from TBI through increase in synaptogenesis and inhibition of inflammation (Dong et al. 2016; Ryskamp et al. 2019).
3. *Nicotinic receptors* Amantadine is an open-channel blocker of α4ß2 nicotinic receptors (Fig. 3, Table 4) with high potency of IC_50_ of 3.44 µM (Buisson and Bertrand 1998). It also shows blocking properties at α7 nicotinic receptors with IC_50_ of 6.5 µM (Albuquerque et al. 1998; Matsubayashi et al. 1997). In general, it is difficult to explain the beneficial therapeutic effects of amantadine by nicotinic receptor antagonism (Dineley et al. 2015; Tizabi and Getachew 2017) except for antidyskinetic activity and anti-inflammatory effects. Mecamylamine, an α3ß4 receptor antagonist which seems to block α4ß2 receptors, produces antidyskinetic effect in hemiparkinsonian rats (Bordia et al. 2010), however, some other authors did not observe such effect in this model (Dekundy et al. 2007). Furthermore, similar effects were found after nicotine agonists, but were attributed to receptor desensitization effect (Bordia et al. 2010).
4. *Phosphodiesterase (PDE)* Amantadine inhibits calmodulin-dependent phosphodiesterase 1 (PDE1, Fig. 3, Table 4) with IC_50_ of ca. 5 µM which may increase adenosine 3′,5′-cyclic monophosphate (cAMP) and in turn produce neuroprotective activity (Kakkar et al. 1997) and connected anti-inflammatory properties of amantadine (O'Brien et al. 2020). In another study, in vitro amantadine at concentration of 6 µM inhibited PDEs responsible for guanosine 3′,5′-cyclic monophosphate (cGMP) and cAMP degradation by up to 30 and 20%, respectively (Sancesario et al. 2014). This effect analyzed ex vivo was stronger in dyskinetic animals reaching 50% effect. Moreover, amantadine treatment (40 mg/kg) decreased cGMP in the striatum of dyskinetic animals as evidenced by brain microdialysis (Sancesario et al. 2014). D1 receptor functional super-sensitivity, abnormal modulation of cAMP cascade, and enhanced dopamine- and cAMP-regulated phosphoprotein kDa (DARPP-32) phosphorylation have been suggested as the most plausible long-standing mechanism of l-DOPA dyskinesias (Feyder et al. 2011). There is an indication that PDEs may be upregulated in TBI and some PDEs, particularly from group 4, have been proposed as possible treatments (Titus et al. 2014; Wilson et al. 2016).
5. *Glial-cell-derived neurotrophic factor (GDNF)* In vitro, amantadine produced a stronger increase in GDNF (Fig. 3, Table 4) mRNA than memantine (both at 5 µM) (Caumont et al. 2006). Effect on release was seen with EC_50_ of 6.2 µM, while memantine which is approx. 20 times more potent at NMDA receptors had similar or lower potency (Caumont et al. 2006). In vitro, amantadine reduced neuronal toxicity produced by lipopolysaccharide (LPS) and 1-methyl-4-phenylpyridinium (MPP^+^, ca. 10–20 µM) presumably through decrease in inflammation and increase in GDNF mRNA production in astroglia (Ossola et al. 2011). Amantadine in rats given at 10 mg/kg for 2 weeks increased GDNF mRNA expression in cortex and hippocampus (Rogoz et al. 2007). It was suggested that amantadine may increase mRNA GDNF expression by inducing the acetylation of histone H3 and/or by inhibiting the histone deacetylase (Ossola et al. 2011). In another study, amantadine given for 3 days in rats at the dose of 25 mg/kg increased GDNF on the protein level (Zhang et al. 2014) and improved recovery after postoperative insult. Another study showed that exercise produced antidyskinetic effect which was associated with increase in BDNF expression (Speck et al. 2019). Amantadine (60 mg/kg) also attenuated dyskinesia, but did not produce an additive effect to exercise suggesting similar mechanisms (ibid). It should be added that NMDA receptors do not seem to be involved in this neuroprotective effect of amantadine. GDNF seems also to be involved in alleviation of postoperative cognitive dysfunction in rats by amantadine given at low dose of 25 mg/kg (Zhang et al. 2014; Zhong et al. 2020). Effect on GDNF could potentially improve L-DOPA-induced dyskinesia (Speck et al. 2019) and afford neuroprotection in Parkinson’s disease, Huntington’s disease, ALS, and other disorders involving motor neuron neurodegeneration (Allen et al. 2013; Biju et al. 2010; Cheng et al. 2018; Lapchak 1996). Clearly, an increase in GDNF would be supportive in recovery after TBI (Minnich et al. 2010). Based on preclinical studies, it has been suggested that amantadine may produce also antidepressant effect through GDNF (Tsybko et al. 2017).

**Fig. 2:**
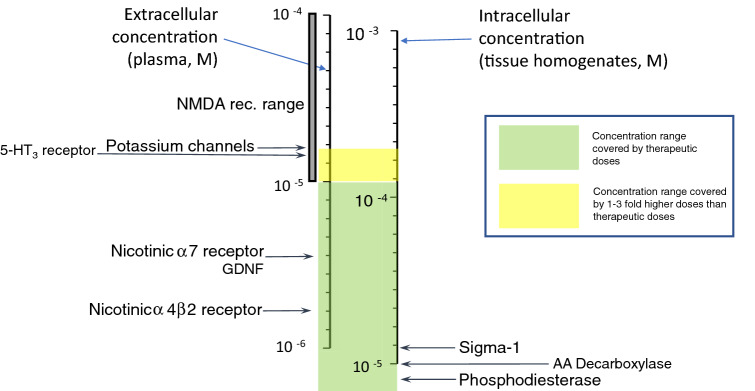
Graphic presentation of known in vitro actions of amantadine positioned on the concentration scale in relation to therapeutic levels. On the left extracellular targets are shown while on the right intracellular

There are also several targets which are likely affected by concentrations 1- to 3-fold above the range achieved after administration of amantadine at clinically used doses and which are defined as “possible targets” (see Table 4):

1. *NMDA receptors* Although the majority of the in vitro studies investigating the effect of amantadine on NMDA receptors show effects with IC_50_ values above plasma therapeutic range of 10 µM (Fig. 2, Tables 3, 4); nevertheless, this action should not be neglected and may have supportive potential as add-on to other actions.
2. *Serotonergic (5-HT) receptors type 5-HT3* Ondansetron (5-HT3 antagonist) has been shown to inhibit dyskinesia-like behavior in rats treated with L-DOPA (Aboulghasemi et al. 2019). Moreover, 5-HT3 antagonism has been proposed as a therapeutic approach for a number of indications such as depression, emesis, irritable bowel syndrome (IBS), schizophrenia, anxiety, cognitive deficit, pruritis, inflammation, and pain (Thompson and Lummis 2007)
3. *Potassium channels* At drug concentrations approximately three times higher than therapeutically relevant (Table 4), amantadine blocks inwardly rectifying potassium channels (Kir2) that control the intrinsic excitability of GABAergic spiny projection neurons (SPNs, IC_50_ = 27 µm), without significantly diminishing synaptic NMDA currents (Shen et al. 2020). These effects were implied to contribute to the antidyskinetic effects of amantadine following l-DOPA treatment and possibly in pain (Bhave et al. 2010; Shen et al. 2020).

**Fig. 3:**
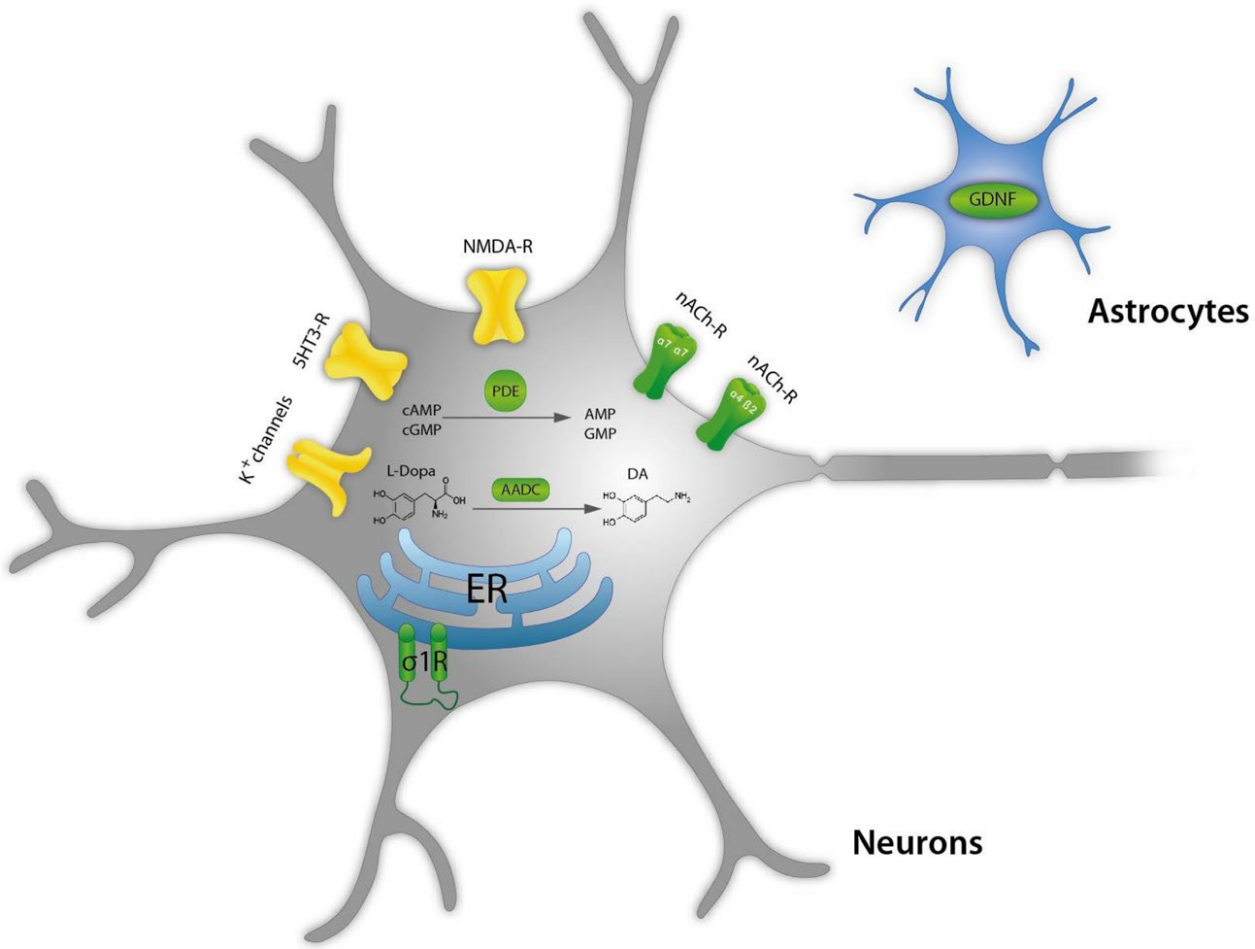
Scheme of cellular location of amantadine targets. Likely targets are in green and possible targets in yellow

These targets are graphically shown in Fig. 2 in relation to their affinity and additionally according to their location in Fig. 3.

It should also be mentioned that Moresco and colleagues observed that amantadine treatment for 10–14 days at 200 mg produced in patients an enhancement (ca. 10%) in [^11^C-]raclopride binding indicating an increase in dopaminergic 2 (D2) receptors which may be involved in antiparkinsonian activity (Moresco et al. 2002). This is most probably consequence of one or several actions listed in Table 4. Similarly, the indirect DA-mimetic effect could increase arousal in comatose patients (Sawyer et al. 2008). Amantadine, in addition to its weak NMDA antagonist properties, has been demonstrated to increase extracellular DA concentrations by blocking its reuptake and facilitating its synthesis (Baldessarini et al. 1972; Brown and Redfern 1976; Gianutsos et al. 1985; Von Voigtlander and Moore 1971). Moreover, the drug has been shown to increase density (Gianutsos et al. 1985) or changing the conformation (Allen 1983) of postsynaptic DA receptors. In summary, DA-ergic probably indirect actions of amantadine comprise presynaptic and postsynaptic effects (Meythaler et al. 2002).

Similarly, anti-inflammatory effects described for amantadine below may be secondary:

1. Using cultured microglial cells, it was demonstrated that the drug inhibited inflammatory activation of microglia by ca. 25% at 4 µM concentration and a signaling pathway that governs the microglial activation following LPS stimulation. Moreover, at 49 µM, it protected neurons in co-culture (Kim et al. 2012).
2. In vitro, amantadine at 1 µM inhibited by 30% production of inflammatory cytokines such as interferon-γ (IFN-γ) and tumor necrosis factor-α (TNF-α) in human blood (Kubera et al. 2009).
3. In vivo in mice amantadine (10 mg/kg) given for 4 days inhibited microglia activation and at 25 mg/kg provided protection against MPTP (Kim et al. 2012).
4. Amantadine stimulates production of interleukins in humans (Wandinger et al. 1999).

## Preclinical and clinical evaluation of amantadine’s non-canonical indications

### Neuroprotection and disease modification: general aspects

While there is only limited knowledge about specific causal mechanisms underlying neurodegenerative diseases, there is accumulating evidence that oxidative stress, excitotoxicity, calcium-dependent cell death, dysfunction of key proteins, lysosomal and autophagy dysfunction, etc. are responsible for the degeneration of nerve cells. However, there is lack of knowledge as to the follow-up of such processes and their interaction in the various stages of disease process makes it very difficult to develop specific neuroprotection. Therefore, a more recent strategy is to develop drugs, which may lead to “disease modification”, meaning that they influence the disease process in slowing the degenerative progression.

Amantadine has shown protective properties in several experimental studies. For example, it reduced activation of microglia, induced expression of GDNF in astroglia in primary cultures with different composition of neurons, microglia, and astroglia (Ossola et al. 2011). Furthermore, amantadine in vitro showed antioxidative activity in the 2,2-diphenyl-1-picrylhydrazyl (DPPH) assay (Kranthi et al. 2019). In rats, it attenuated the loss of nucleus basalis magnocellularis cholinergic cells induced by NMDA injected directly into this region (Wenk et al. 1995). In the same model, amantadine neuroprotection was provided by amantadine infusions at 100 mg/kg/day as evidenced by cortical choline acetyl transferase (ChAT) activity (Wenk et al. 1996). Furthermore, amantadine prevented a decrease of striatal homovanillic acid concentration induced by 1-methyl-4-phenyl-1,2,3,6-tetrahydropyridine (MPTP) treatment in mice (Rojas et al. 1993).

Amantadine blocked 1-methyl-4-phenylpyridinium (MPP^+^) cytotoxicity involving noradrenaline (NA) transporter starting with 30 µM, while ketamine and memantine were not protective up to 100 µM (Sommerauer et al. 2012). In cells expressing DA uptake or NMDA receptors, protective effect of amantadine against MPP^+^ was weaker (Sommerauer et al. 2012). The lower potency of amantadine in cytotoxicity assays on NR1/2A receptor-expressing cells as compared to patch-clamp recordings can be explained by the absence of Mg^2+^ in the electrophysiological experiments, whereas cytotoxicity assays were performed in cell culture medium with Mg^2+^ concentrations in the low millimolar range as to be expected in vivo.

Some support for potential neuroprotective action of amantadine came from retrospective analysis of survival which suggests increased life expectancy of Parkinsonian patients treated with amantadine (Uitti et al. 1996).

In conclusion, amantadine in experimental studies shows neuroprotective properties related to antioxidative, anti-inflammatory, and molecular mechanisms. These properties at a cellular level could be secondary to amantadine’s complex MoA.

### Infections with CNS involvement: neuroprotection

#### Preclinical studies

The antiviral potency of aminoadamantanes discovered over 5 decades ago (Davies et al. 1964; Gerzon et al. 1963) has been attributed to inhibition of virus replication (Kendal and Klenk 1991; Tanner et al. 2005). These drugs recently regained again focus of research and drug development (Kesel et al. 2012) due to their possible potential in the COVID-19 pandemic (Brison et al. 2014; Butterworth 2020c; Riederer and Ter Meulen 2020). In the present review, we decided not to discuss the direct effects of amantadine on the virus, since several reviews on that topic have been published already elsewhere (Aranda-Abreu et al. 2020c; Brenner 2020; Butterworth 2020b; Smieszek et al. 2020; Tipton and Wszolek 2020). However, we discuss shortly below the protective actions of amantadine on nervous system against viral infections.

Brison and colleagues clearly demonstrated that S-mutant HCoV-OC43 infection in mice caused glutamate excitotoxicity expressed as dysregulation of glutamate recycling via the glial transporter-1-protein and glutamine synthase (Brison et al. 2014). In the same study, memantine—a derivative of amantadine—improved clinical scores related to paralytic disease and motor disabilities. Of interest is the notion that memantine also reduced HCoV-OC43 replication in the CNS in a dose-dependent manner (Brison et al. 2014). To understand the MoA of aminoadamantanes as anti-SARS-CoV-2 agents, Abreu et al. (2020) proposed that amantadine blocks the viroporin E channel of SARS-CoV-2, thus preventing the release of the viral nucleus into the cell cytoplasm (Aranda-Abreu et al. 2020c). Using docking models, Abreu et al. (2020) demonstrated that the ligand site of amantadine could interact with the PHE26 amino acids of the alpha helix. This work is based on earlier research (Evans and Havlik 1994; Jimenez-Guardeno et al. 2014; Thomaston et al. 2018; Torres et al. 2007; Wang et al. 1993). The mechanism of action could apart from above mentioned blockade of viroporins involve also action at lysosomes (Brenner 2020; Smieszek et al. 2020), some other yet unknown effect, or a combination thereof.

#### Human studies

Amantadine has been used as an antiviral agent and it readily crosses the blood–brain barrier, making the drug a potential candidate for the treatment of viral infections affecting the central nervous system. Notwithstanding, clinical data regarding the effects of amantadine on neurological symptoms in course of viral infections remain scarce. There was a limited early evidence that it may be treating the prion-induced Creutzfeldt–Jakob disease (Sanders 1979). Robertson et al. reviewed 38 cases of subacute sclerosing panencephalitis (SSPE) associated with measles or rubeola infections. These pediatric patients showed a broad array of psychiatric and neurological alterations including but not limited to learning deficits, personality changes, seizures, myoclonus, spasticity, and extrapyramidal dysfunction. Twenty four of the 38 patients died; 15 received antiviral treatment with amantadine or ribavirin. Of the two drugs, only amantadine apparently increased survival duration and led to prolonged remissions (Robertson et al. 1980). Amantadine studies comprise partly conflicting case reports, e.g., for Borna virus infection (Bode et al. 1997; Stitz et al. 1998). On the other hand, some evidence can be found on beneficial effects of amantadine on some psychiatric symptoms associated with neurotropic virus infections (see the Sect. 5.11).

In confirmed COVID-19 patients suffering from MS, PD and cognitive impairment treated with amantadine (100 mg QD) or memantine (10 mg BID) did not develop clinical manifestations of infectious disease (Rejdak and Grieb 2020). There are also two low-powered studies suggesting a beneficial effect of amantadine in SARS-CoV-2-infected patients (Aranda-Abreu et al. 2020a; Cortes Borra 2020). If confirmed with higher numbers of patients, amantadine may add to the therapeutic armamentarium fighting SARS-CoV-2 infection.

However, in the available literature, there are no relevant randomized clinical trials specifically addressing the neuroprotection by amantadine in viral infections or its effects on COVID-19 symptoms.

### Traumatic brain injury (TBI): recovery enhancement

#### Preclinical studies

Wang and colleagues (Wang et al. 2014) administered amantadine for 1 h after TBI followed by dosing three times daily for 16 consecutive days at 15, 45, and 135 mg/kg/day. The highest dose improved Morris maze spatial learning and provided neuroprotection in the hippocampus (Wang et al. 2014). However, the effective dose produced 12,000 ng/ml (ca. 80 µM) serum levels which are far above therapeutic range (see Table 3).

After cortical impact injury, treatment with amantadine (20 mg/kg) starting 24 h after insult for 19 days improved motor and learning functions tested 24 h after the last amantadine dose, but failed to decrease cortical lesion volume (Bleimeister et al. 2019). There was no additive effect with enriched environment.

Amantadine (10 mg/kg/day for 18 days) treatment starting 1 day following TBI improved recovery as evidenced by water maze performance tested on days 14–18 after injury (Dixon et al. 1999). Motor performance (beam walking and beam balance) and hippocampal neuron survival were not improved.

In cerebral cortical fluid percussion injury in rats, infusion of amantadine at 86.4 mg/kg/day starting at day 5 for 8 weeks reversed dopamine release deficit and improved motor performance on rotarod and learning in novel object recognition test (Huang et al. 2014).

In the study assessing restorative effect in rats, therapeutic benefit could be observed at an amantadine dose of 20 mg/kg, while at 10 and 40 mg/kg, no effect was observed, indicating bell-shaped dose–response curve (Okigbo et al. 2019).

In another study, amantadine given at 45 or 135 mg/kg 3 times a day for 28 days following TBI decreased the neuronal degeneration and apoptosis in the substantia nigra (Tan et al. 2015). Amantadine also reversed the decrease of dopamine in the striatum and decreased depressive-like behavior (forced swim test, sucrose preference) and learning deficit induced by TBI (Tan et al. 2015; Wang et al. 2014). It should be stressed that even the low dose (45 mg/kg) amounted to daily dose of 135 mg/kg/day which is very high. In turn, translational value of this particular study is questionable as such high plasma (ca. 64 µM) levels cannot be achieved in humans (Wang et al. 2014), see also Tables 2 and 3.

Nevertheless, given all above-discussed studies, preclinical evidence suggests the utility of amantadine in post-treatment in TBI to improve recovery. This is also supported by several clinical studies (see next section).

#### Human studies

In their classical manual “Diagnosis and Treatment of Stupor and Coma”, Plum and Posner defined coma as a pathology marked by severe and prolonged dysfunction of vigilance and consciousness (Plum and Posner 1972). More recently, consciousness has been described as a combination of arousal (i.e., wakefulness, sustained attention, or vigilance, clinically determinable by the presence of eye opening) and awareness (comprising all subjective perceptions, feelings, and thoughts) (Posner et al. 2007; Zeman 2001). The level of arousal is maintained by the brainstem and the thalamus (Lin 2000; Schiff 2008). Normal vigilance requires preserved reticular activating system (RAS) in the upper pons, as well as the intralaminar nucleus of the thalamus, involved in filtering and integrating sensory inputs (Buckwalter et al. 2008; Sherman and Guillery 2002). These structures cooperate with some fronto-parietal regions shown to be affected in patients with impaired consciousness (Laureys 2005; Laureys et al. 1999, 2000). There are two primary mechanisms leading to coma: a diffuse bilateral insult to cerebral hemispheres or a focal coma comprise stroke, trauma, and drug overdose. On a neurochemical level, DA is thought to play a major role in arousal and in the TBI, as widespread damage to axons in the brain is associated with a reduction of DA availability (Bales et al. 2009; Meythaler et al. 2002).

Studies with amantadine in patients with disorders of consciousness are heterogenous in terms of outcome measures and populations studied. The available evidence suggests, however, beneficial effects of amantadine in the recovery of patients with acute TBI (Ma and Zafonte 2020). An early placebo-controlled crossover randomized-controlled trial (RCT) of 10 subjects with moderate-to-severe TBI found no difference in the rate of cognitive recovery with amantadine (Schneider et al. 1999). However, a later RCT demonstrated that patients having received amantadine improved compared to those receiving placebo on the Disability Rating Scale (DRS) involving sustained attention score, and further tests of cognitive function. Patients in the placebo group improved further when switched to amantadine (Meythaler et al. 2002). A more recent large-sample (*n* = 184) placebo-controlled RCT of amantadine involved patients 4–16 weeks after severe TBI presenting as the vegetative state or minimally conscious state demonstrated faster recovery on the DRS and Coma Recovery Scale-Revised (CRS-R) in an active-treatment group during the 4-week treatment period (Giacino et al. 2012). The improvements were maintained during a 2-week wash-out period in both groups, but its rate decreased in the amantadine group, so that no difference in DRS and CRS-R scores was found between the active and placebo groups 6 weeks. Both groups were similar in terms of adverse effects (Giacino et al. 2012).

The results of the RCTs in patients with disorders of consciousness are, in general, in agreement with other studies of various designs, e.g., retrospective chart reviews, case–control studies, or case reports (Chandler et al. 1988; Gualtieri et al. 1989; Kraus and Maki 1997a, b; Kraus et al. 2005; Nickels et al. 1994; Raffaele et al. 2002; Saniova et al. 2004, 2006; Whyte et al. 2005; Wu and Garmel 2005; Zafonte et al. 2001, 1998). Amantadine treatment was also demonstrated to produce specific metabolic changes in affected brain areas, which correlated with some improvements in TBI patients (Kraus et al. 2005; Schnakers et al. 2008).

Amantadine was also relatively widely tested in pediatric populations with TBI. McMahon et al. (2009) compared the drug with placebo in a crossover RCT in children (*n* = 7). Although there were no differences between the active and placebo groups, there were greater improvements in consciousness observed with amantadine (McMahon et al. 2009). Furthermore, Patrick et al. (2006) compared amantadine to pramipexole in an RCT in children and adolescents in a low-responsive state 1 month post-injury. Patients in both groups made significant improvements on the Coma/Near Coma Scale, the Western NeuroSensory Stimulation Profile, the DRS weekly gains, and Rancho Los Amigos Scale. There were no significant side effects to treatment (Patrick et al. 2006). In a study by Green et al. (2004) evaluating the safety of amantadine in a pediatric population, only 5 of 54 patients experienced side effects that were readily reversible (Green et al. 2004). Beers et al. (2005) studied the effects of amantadine in pediatric patients after TBI and found the medication safe. Even though the differences in cognition were not found statistically significant, the authors suggested a potential cognition improvement with amantadine (Beers et al. 2005).

Even though amantadine failed to produce favorable effects in some of the studies conducted [e.g., (Hughes et al. 2005; Schneider et al. 1999)], it can be concluded that there is some evidence that amantadine safely improves arousal and some aspects of cognitive function (e.g., attention, concentration, alertness, arousal, and mobility) in comatose patients with acute brain injury at different stages (DeMarchi et al. 2005; Sawyer et al. 2008). This is reflected by several recent clinical practice guidelines (Anghinah et al. 2018; Butterworth 2020a; Plantier and Luaute 2016).

### Stroke

#### Preclinical studies

In a rat model of ischemia based on the middle brain artery occlusion, amantadine sulfate produced enhancement of heat shock protein 70 (HSP 70) expression indicative of the activity of preventive mechanisms (Khasanova et al. 2009). Two other studies showed that amantadine at low dose of 25 mg/kg given after common carotid artery narrowing decreased cognitive deficit in rats (Zhang et al. 2014; Zhong et al. 2020).

#### Human studies

There are several very heterogenous reports on potential effectiveness of amantadine in patients who underwent different kinds of stroke. Khasanova et al. compared the clinical effectiveness of infusions of amantadine (*N* = 20) and magnesium sulfate (*N* = 20) in the acute period of ischemic stroke. Patients treated with amantadine showed more pronounced restoration of consciousness and regression of neurological deficits, observed particularly in the first time of the therapy (Khasanova et al. 2009). Krivonos et al. investigated the effect of co-treatment with amantadine (200 mg in 500 ml i.v. over 3 h for 10 days) plus standard-of-care (*N* = 23) versus standard-of-care therapy (*N* = 10) in patients with ischemic stroke. The therapy was initiated in the majority of patients within 24–48 h of onset. The efficacy was assessed using National Institutes of Health Stroke Scale (NIHSS), the modified Rankin scale, and the Barthel Daily Living Index on in-patient day 10 and 3 months after stroke. On day 10, the group administered amantadine showed a significant 39.1% reduction in neurological deficit on the NIHSS, especially in mild stroke, but regardless of age or stroke subtype; in the corresponding reduction in the control group reached 24.4%. Likewise, after 3 months, the neurological deficit on the NIHSS in the amantadine group decreased by 58.7%, while in the control group by 41.8%. In line with the above, also was a significant reduction in Rankin scale scores were higher in amantadine-treated group than in the group treated with standard-of-care therapy, with significant differences observed at 3 months. Three months after stroke, the Barthel Daily Living Index score increased significantly in both treatment groups, with no significant differences between the groups (Krivonos et al. 2010).

Recently, Akcil et al. (2018) studied the effects of amantadine on neurocognitive function recovery from subarachnoid hemorrhage (SAH) over 6 months. A group of five patients received the standard-of-care plus amantadine for 30 days, while the other seven patients received the standard treatment only on the first and fifth days and at the third and sixth months after admission. The data suggested that adding amantadine to the standard treatment during the early period of SAH may improve neurocognitive function recovery evaluated using the Coma Recovery Scale-Revised (CRS-R) and Disability Rating Scale (DRS) (Akcil et al. 2018).

In the most recent and most extensive retrospective study, Leclerc et al. (2020) reviewed hospital records to evaluate safety and effectiveness on neurostimulants amantadine and/or modafinil (both mostly at the daily dose of 100 mg b.i.d) used for acute stroke care in the intensive-care unit (ICU). The drugs were initiated 1–27 (median 7) days post-stroke. Only patients receiving amantadine monotherapy and/or combination of amantadine and modafinil met the responder definition (2 of 3 criteria within 9 days of neurostimulant initiation: ≥ 3 points increase in Glasgow coma scale (GCS) score from pre-treatment baseline; improved wakefulness; clinical improvement). The median time to response was 3 days, and the responders were more frequently discharged home or to acute rehabilitation than non-responders (Leclerc et al. 2020).

Two very recent studies by Gao et al. add to the already available evidence that amantadine may be useful in the management of sequelae of cerebrovascular events. In a retrospective cohort study, amantadine (100–200 mg/day p.o.) was demonstrated to accelerate recovery (as measured on Glasgow Out Scale 5 months after onset) in patients with persistent vegetative state after severe cerebral hemorrhage scores compared to a matched control cohort (Gao et al. 2020b). In a case-series study, amantadine administration (150–200 mg/day p.o.) showed beneficial effects on neurological recovery of 7 patients after severe cerebral hemorrhage, as evaluated using revised coma recovery scale (CRS-R) recovery (Barrett and Eslinger 2007; Gao et al. 2020a).

Of note, a pilot open-label study investigated verbal fluency on and off amantadine (100 mg BID) in aphasic patients after stroke or other brain insults using Controlled Oral Word Association Test. Amantadine administration significantly improved word generation (Barrett and Eslinger 2007; Gao et al. 2020a).

### Spinal cord injury

#### Preclinical studies

Amantadine (45 mg/kg/day) treatment for 7 days directly following spinal cord injury (SCI, clip compression insult) decreased oxidative stress markers malondialdehyde (MDA) and myeloperoxidase (MPO), increased glutathione (GSH) levels, improved histology, and decreased apoptotic markers (Dogan and Karaca 2020). In turn, it was suggested that a protective effect occurred via stimulation of angiogenesis, decreasing inflammation, oxidative stress, and apoptosis.

This study is in line with TBI experiments showing utility of amantadine in improvement of recovery after insult, and is encouraging enough to follow this indication in further preclinical studies and later with clinical investigations.

#### Human studies

In the publicly available scientific literature, no relevant clinical reports on effects of amantadine in patients with spinal cord injury could be identified.

### Multiple sclerosis (MS)

#### Preclinical studies

In the experimental autoimmune encephalomyelitis (EAE) model in rats (based on injection of Freund's complete adjuvant, CFA), amantadine at the dose of 100 mg/kg/day suppressed significantly disease score and decreased expression of pro-inflammatory cytokines (IL-1β, IL-6, fractalkine, MIP-1, and MIP-3) (Sulkowski et al. 2013). It should be stressed that 100 mg/kg is an amantadine dose which is regarded as slightly above clinically relevant levels. In the follow-up study, in the same model, it was shown that amantadine also partially attenuates increase in glutamate in the brain observed in EAE rats (Sulkowski et al. 2014). It should be added that synaptic morphology was not improved by amantadine in these animals.

In another study in EAE mouse model of MS 40 mg/kg amantadine, applied daily by a feeding cannula, did not suppress the incidence or severity of EAE, but improved significantly the recovery phase. In addition, a significant increase in CD4^+^, CD25^+^, Foxp3^+^, and T cells could be detected in response to amantadine treatment (Fukumoto et al. 2019).

It could be concluded that, so far, there is no strong preclinical evidence that amantadine could be useful in the treatment of pathology of MS in humans.

#### Human studies

Amantadine is one of the pharmacological interventions that were most extensively studied for MS-related fatigue. Already in 1985, Murray et al. carried out a double-blind placebo-controlled trial of amantadine (100 mg BID) in 32 patients with MS and observed improvements in fatigue in 62.5% of patients on amantadine and 21.8% on placebo (Murray et al. 1985). Management of MS-related fatigue with amantadine has been studied in controlled trials, demonstrating improvements in both subjective and objective ratings of fatigue (see Table 5). However, neither amantadine nor other drugs used in MS-related fatigue (i.e., modafinil or methylphenidate) were found superior to placebo in a recent randomized, placebo-controlled, double-blind crossover trial. Furthermore, the use of these drugs was associated with increased rates of adverse events (Nourbakhsh et al. 2020).

**Table 5:** Summary of clinical studies with amantadine for not conventional indications (not for Parkinson’s disease and viral infections)

Indication	References	Description of the disorder	Population, design	Clinical outcome parameters, scales, scores	Amantadine dose, treatment duration	Results
TBI	Nickels et al. (1994)	Acute in-patient rehabilitation following brain injuries	Case series *N* = 12	Functional, neurobehavioral and cognitive status (e.g., attention, concentration, alertness, arousal, reaction time, agitation, and anxiety)	50–200 mg/day BID	Improvements in attention and concentration, alertness, arousal, processing time, psychomotor speed, mobility, vocalization, agitation, anxiety, and participation in therapy
	Kraus and Maki (1997b)	TBI	Case series *N* = 7	The Mini-Mental State Examination (MMSE), Test for Severe Impairment; Clock Drawing Test; The Hopkins Verbal Learning Test; Hopkins Attention Screening Test; The Brief Test of Attention; verbal fluency tests; The Trail Making Test; Boston Naming Test	25–400 mg/day	All patients had significant frontal lobe dysfunction from TBI, and 4 were “responders”, while 3 were “non-responders” to amantadine treatment, with improvements in alertness, attention, executive function, cognition, speech, behavior, mood, motivation, motor abilities, and psychomotor speed, as well as less dyscontrol
	Saniova et al. (2006)	Closed head injury	RCT, Open Label 32 (amantadine * N* = 18)	Glasgow Coma Scale (GCS), survival, biochemical parameters: glycaemia, malondialdehyde (MDA; marker of lipid peroxidation), beta-carotene, total SH groups	200 mg i.v. BID	Amantadine-treated patients had reduced MDA and increased beta-carotene (antioxidant), as well as improved survival, after only 1 week of treatment
	Green et al. (2004)	TBI (pediatric)	Case–control, Retrospective *N* = 118 (amantadine * N* = 54)	Ranchos Los Amigos (RLA)	100 mg BID to 400 mg QD	Amantadine-treated subjects had a greater improvement in their RLA level during their admission. Subjective improvements noted in most patients administered amantadine. Side effects were minimal and resolved when treatment was reduced
	Hughes et al. (2005)	Severe TBI	Retrospective Cohort *N* = 123 (amantadine * N* = 28)	GCS and somatosensory evoked potentials	200 mg BID	Amantadine failed to shorten the time to emerge from coma
	Patrick et al. (2006)	TBI	RCT *N* = 10 (amantadine * N* = 6)	Coma Near Coma (CNC) scale, DRS, and Western NeuroSensory Stimulation Profile	100 mg BID	Weekly rate of change in the CNC scale, DRS, and Western NeuroSensory Stimulation Profile was significantly greater with amantadine or pramipexole than without and slowed 6 weeks after treatment termination)
	Whyte et al. (2005)	TBI	Cohort *N* = 124 (amantadine * N* = 47)	Disability Rating Scale (DRS)	Not provided	Amantadine significantly improved recovery
	Vargus-Adams et al. (2010)	Brain injuries in pediatric population	RCT, crossover *N* = 7	Coma Near Coma (CNC) Scale or Coma Recovery Scale-Revised (CRS-R)	400 mg/day	Amantadine was well tolerated, but had no significant effect on CNC Scale or CRS-R
	Giacino et al. (2012)	Post-traumatic disorders of consciousness	RCT, crossover *N* = 184 (amantadine * N* = 87)	Functional recovery	200 mg BID	Amantadine accelerated the rate of functional recovery during active treatment. Amantadine did not increase the incidence of adverse effects
	Ghalaenovi et al. (2018)	Severe TBI	RCT *N* = 40 (amantadine * N* = 19)	Glasgow–Coma Scale (GCS)	100 mg BID	Patients having received amantadine had a faster rate of improvement in their GCS scores during the first week of treatment. No functional differences were observed at 6-month follow-up
	Hammond et al. (2014)	TBI	RCT *N* = 76 (amantadine * N* = 38)	Neuropsychiatric Inventory-Irritability (NPI-I); Neuropsychiatric Inventory-Aggression (NPI-A)	100 mg BID, 4 weeks	Among patients with moderate-severe irritability (≥ 6 months following TBI), 4 weeks of amantadine significantly improved the frequency and severity of irritability and aggression and was safe
	Hammond et al. (2015)	TBI	RCT *N* = 168 (amantadine * N* = 82)	Neuropsychiatric Inventory-Irritability (NPI)	100 mg BID	Because of a very large placebo effect, amantadine did not significantly improve irritability (in patients with moderate-severe irritability, who suffered TBI ≥ 6 months prior to enrollment)
	Gramish et al. (2017)	TBI	Cohort, retrospective *N* = 139 (amantadine * N* = 70)	Agitation, length of stay in intensive-care unit (ICU)	100 mg BID	Agitation was significantly more prevalent in the amantadine group. Patients given amantadine had longer ICU lengths of stay and received more opioids
	Hammond et al. (2015)	TBI	RCT *N* = 118 (amantadine * N* = 61)	Aggression, anger	100 mg BID	Among patients (≥ 6 months post-TBI) with moderate-to-severe aggression, amantadine significantly reduced aggression, with no beneficial effect on anger
	Schneider et al. (1999)	TBI	RCT, Crossover *N* = 10 2 weeks on AMH, 2 weeks wash-out, 2 weeks on placebo	Neurobehavioural Rating Score (NRS) Orientation, memory, attention, executive Rate of patients’ cognitive recovery	50–150 mg BID over 2 weeks	Amantadine had no effect on the rate of patients’ cognitive recovery. Results limited by small sample size, heterogeneous population, acute time course, and limited study power, and high drop-out rate
	Meythaler et al. (2002)	Acute TBI	RCT, crossover *N* = 35 6 weeks on AMH, 6 weeks on placebo	Agitated Behavioural Scale (ABS); Mini-Mental Status Examination (MMSE); Disability Rating Scale (DRS); Glasgow Outcome Scale (GOS); and Functional Independence Measure (FIM-cog) scale; Galveston Orientation and Amnesia Test (GOAT)	200 mg/day over 6 weeks	Significant improvements in the MMSE, DRS, GOS, and FIM cognitive scale in both groups of patients recovering from acute TBI during the first 6 weeks of the study, but only in the amantadine-treatment group during the second 6 weeks. However, the groups had similar functional levels after the study had finished Amantadine was safe in the study population
	Raffaele et al. (2002)	Brain injuries	RCT, crossover *N* = 6	Attention and concentration, fatigue	Up to 150 mg BID	Amantadine improved attention and concentration, and reduced fatigue
	Beers et al. (2005)	TBI (pediatric subjects)	RCT (BUT: no placebo) *N* = 27 (amantadine * N* = 17); Only per-protocol set analyzed: * N* = 13 (amantadine * N* = 9)	Cognition	Up to 150 mg/day (< 10 y/o) or 200 mg/day (> 10 y/o)	Improvements with amantadine in cognitive testing when compared to age- and severity-matched TBI control patients observed in those ≤ 2 years post-injury. The results were limited, since just per-protocol analysis was used
	Kraus et al. (2005)	TBI	RCT, open label, crossover *N* = 22	Executive function	400 mg/day	Amantadine improved performance on executive function tests, correlated with a significant increase in left prefrontal cortex glucose metabolism in the first 6 male subjects enrolled
	Reddy et al. (2013)	Subjects with history of head concussion –	Case–control, retrospective *N* = 50 (amantadine * N* = 25)	Verbal memory, reaction time	100 mg BID	After 3–4 weeks, amantadine-treated patients made significantly greater improvements in verbal memory and reaction time, as well as reported fewer persistent post-concussion symptoms, when compared to matched controls (by age, sex, and concussion history)
	Hammond et al. (2018)	TBI (at least 6 months prior to enrollment, with moderate-severe irritability)	RCT *N* = 119 (amantadine * N* = 59)	Cognitive battery, irritability	100 mg BID	No differences between groups were observed after 60 days of treatment, but the placebo responses were high. Cognitive battery baseline scores for the treatment group were higher, increasing the group’s susceptibility to ceiling effects. At day 28, the mean change for the placebo group was greater
Stroke	Barrett and Eslinger (2007)	Stroke (3), postoperative injury	Case Series *N* = 4	Aphasia (Controlled Oral Word Association test)	100 mg BID	Patients with transcortical motor aphasia performed better on Controlled Oral Word Association test during the 6 days when given amantadine
	Akcil et al. (2018)	Stroke (due to aneurysmal subarachnoid hemorrhage)	RCT *N* = 12 (amantadine * N* = 5)	Coma Recovery Scale-Revised (CRS-R), Disability Rating Scale (DRS)	200 mg/day or 100 mg BID	At both 5 days and 6 months after intensive-care unit admission, patients (who suffered aneurysmal subarachnoid hemorrhage) who were given amantadine had higher CRS-R and lower DRS scores
	Khasanova et al. (2009)	Stroke (atherothrombotic or cardioembolic, acute period)	*N* = 40 (amantadine (PK-Merz) = 20, magnesium sulfate control)	Consciousness, neurological deficits	200 mg (in 500 ml) i.v. infusion	Patients treated with amantadine sulfate exhibited the more significant restoration of consciousness and better dynamics (regress) of neurological deficit with the most intensive restoration of neurological deficit in the first day that allows to recommend the use of amantadine sulfate in the first hours of ischemic stroke and for the prevention of reperfusion damage in recanalisation therapy of ischemic stroke
	Krivonos et al. (2010)	Acute ischemic stroke (within 24–48 h of onset)	*N* = 33 (amantadine * N* = 23) 10 patients in the control group received standard-of-care treatment	Severity of Neurological Deficit on the National Institutes of Health Stroke Scale (NIHSS), the modified Rankin scale, and the Bartel Daily Living Index	200 mg (500 ml) i.v. over 3 h 10 days	The results obtained here demonstrated the efficacy and safety of treatment with the glutamate NMDA receptor antagonist amantadine sulfate administered during the acute period of stroke. It is particularly indicated in patients with mild to moderate neurological deficit
	Leclerc et al. (2020)	Stroke (47% hemorrhagic, 33% ischemic, 20% subarachnoid hemorrhage)	*N* = 87 Amantadine in * N* = 71 (82%) Modafinil * N* = 13 (15%) or both in 3 (3%) patients	Somnolence, following commands, eye opening, Glasgow coma scale (GCS) score	100 mg BID (* N* = 61; 86%), 100 mg QD (* N* = 6; 8%), 200 mg QD (* N* = 1; 1%)	Neurostimulants were initiated a median of 7 (4.25, 12.75) days post-stroke (range 1–27 days) for somnolence (77%), not following commands (32%), lack of eye opening (28%), or low GCS (17%). The most common starting dose was 100 mg twice daily for both amantadine (86%) and modafinil (54%). Of the 79 patients included in the effectiveness evaluation, 42 (53%) were considered responders, including 34/62 (55%) receiving amantadine monotherapy and 8/24 (33%) receiving both amantadine and modafinil at the time they met the definition of a responder. No patient receiving modafinil monotherapy was considered a responder. The median time from initiation to response was 3 (2, 5) days. Responders were more frequently discharged home or to acute rehabilitation compared to non-responders (90% vs 62%, *p* = 0.006). Among survivors, 63/72 (88%) were prescribed a neurostimulant at hospital discharge. The most common potential adverse drug effect was sleep disruption (16%)
Neuroleptic-induced weight gain	Deberdt et al. (2005)	Schizophrenia, bipolar disorder	RCT, double-blind * N* = 125 (amantadine * N* = 60)	Change in body weight	100–300 (236 mg on average) mg over 16 weeks	Significantly reduced body weight with amantadine
	Graham et al. (2005)	Schizophrenia, schizoaffective disorder, bipolar disorder	RCT, double-blind * N* = 21 (amantadine * N* = 12)	Change in body weight	up to 300 mg over 12 weeks	Significantly reduced body weight with amantadine
	Ji et al. (2014)	Schizophrenia	RCT, double-blind * N* = 61 (amantadine * N* = 31)	Change in body weight	100–200 mg over 8 weeks	Significantly reduced body weight with amantadine
	Pappa et al. (2010)	Schizophrenia	RCT, double-blind, crossover *N* = 22 (amantadine * N* = 11)	Change in body weight	400 mg over 2 weeks	Significantly reduced body weight with amantadine
	Silver et al. (2005)	Schizophrenia, schizoaffective disorder	RCT, double-blind, crossover *N* = 36 (amantadine * N* = 18)	Change in body weight	200 mg over 3 weeks	Significantly reduced body weight with amantadine
Pain	Amin and Sturrock (2003)	Diabetic neuropathy	RCT *N* = 17	The Neuropathy Symptom Score (NSS), together with visual analogue scale (VAS) pretherapy and 1 wk later VAS-P was repeated together with a VAS used to assess relief in pain (VAS-R) and the Physicians Global Evaluation (PGE) score used to assess response to therapy	200 mg (i.v. infusion)	Amantadine reduced the pain of painful peripheral neuropathy (effect sustained for at least 1 week after the infusion
	Medrik-Goldberg et al. (1999)	Sciatica	RCT lidocaine (5 mg/kg), and a placebo *N* = 30	Spontaneous pain VAS and evoked pain (straight leg raise) every 30 min for 3 h	2.5 mg/kg (i.v. infusion over 2 h)	Lidocaine reduced spontaneous and evoked sciatic pain vs. amantadine or placebo. Maximal pain reduction from the baseline with amantadine was 7%. Straight leg raise test also significantly improved with lidocaine
	Pud et al. (1998)	Surgical neuropathic cancer pain	RCT *N* = 15 vs. Placebo	Spontaneous and evoked pain were measured for 48 h before treatment, during treatment, and for 48 h following treatment. An average pain reduction of 85% was recorded at the end of AMAN infusion vs. 45% following placebo administration	200 mg (infusion in 500 ml) over a 3 h	The difference in pain relief between amantadine and placebo was statistically significant. Mean pain intensity remained significantly lower during the 48 h following amantadine than during 48 h before. Amantadine reduced “wind up”-like pain in 4 patients
	Galbraith (1973)	Pain associated with acute herpes zoster infection	RCT, double-blind, vs. Placebo *N* = 100 (amantadine * N* = 54)	duration of pain up to 28 days and after 28 days	100 mg BID, 28 days	No difference between amantadine and placebo in duration of pain up to 28 days observation period. Pain duration* N* > 28 days in a greater proportion of placebo than amantadine patients
Huntington’s disease	Verhagen Metman et al. (2002)	Huntington disease with hyperkinesias	RCT, double-blind, crossover vs. Placebo *N* = 24 (22 evaluable)	Unified Huntington’s Disease Rating Scale (UHDRS) motor score measuring chorea severity for 7 body areas on a scale from 0 to 4 (maximum total score of 28)	100 mg QID over 14 days	A 36% reduction in extremity chorea with amantadine (400 mg) in all evaluable patients (* N* = 22) and a 56% reduction in highest plasma (* N* = 10)
	O'Suilleabhain and Dewey (2003)	Huntington disease with hyperkinesias	RCT, double-blind, crossover vs. Placebo *N* = 28 (25 randomized, 24 evaluable)	24-point chorea scale validated within the study, subjective assessment of chorea by patients, Quality of Life	100 mg TID over 14 days	Amantadine had no significant effect over placebo on the primary variable. Nineteen (19) subjects on amantadine and only 6 subjects on placebo reported improvements in chorea. Quality-of-life scores improved significantly versus placebo after amantadine treatment
	Lucetti et al. (2003)	Huntington disease with hyperkinesias	RCT, acute crossover vs. placebo followed by open-label extension (* N* = 9)	Modified abnormal involuntary movement scale (AIMS)	200 mg (i.v. infusion in 500 ml saline) over 2 h) vs or placebo (acute blinded phase) 100 mg TID (p.o.) over 1 year (chronic open phase)	A significant reduction of dyskinesia scores during both i.v. and oral amantadine sulfate treatment
Multiple sclerosis	Hader et al. (1987)	Multiple sclerosis with fatigue (mean duration 7.8 years)	RCT, Crossover vs. placebo *N* = 115	Visual Analogue Scale (VAS)	100 mg BID 10 weeks	No significant difference between treatment groups (similar significant improvements with both amantadine and placebo)
	Nourbakhsh et al. (2020)	Multiple sclerosis with fatigue (mean duration)	RCT, double-blind, crossover, vs. placebo *N* = 127 (in addition to amantadine, methylphenidate, and modafinil investigated)	Modified Fatigue Impact Scale (MFIS)—primary efficacy Neuro-QoL, fatigue item bank, and the Epwroth Sleepiness Scale (ESS)	< 100 mg BID (p.o.)	Neither drug was superior to placebo in improving fatigue Adverse events were more frequent with drugs than with placebo
	Krupp et al. (1995)	Multiple sclerosis with fatigue (mean duration 11 years)	RCT, Parallel, 200 g amantadine vs. placebo 56,25 mg pemoline vs. placebo *N* = 93	Fatigue Severity Scale (FSS), MS-Specific Fatigue Scale (MS-FS)	100 mg BID 6/8 weeks	No significant improvement on FSS in the pemoline group. Significant improvement on MS-FS in amantadine group
	Geisler et al. (1996)	Multiple sclerosis with fatigue (mean duration 14.5 years)	RCT, Parallel *N* = 45 (amantadine * N* = 16) Amantadine vs. placebo (* N* = 16) or pemoline (* N* = 139	Fatigue Severity Scale (FSS)	100 mg BID 6 weeks	All three treatment groups improved on tests of attention, verbal memory, and motor speed. No differences between amantadine, pemoline, and placebo
	Tomassini et al. (2004)	Multiple sclerosis with fatigue (mean duration 10.2 years)	RCT, Crossover, vs. carnitine (2 g) *N* = 36	Fatigue Severity Scale (FSS)	100 mg BID 12 weeks per treatment, 12 weeks wash-out	Significant improvement in carnitine group as compared to amantadine
	Ashtari et al. (2009)	Multiple sclerosis with fatigue (mean duration 5.7 years)	RCT, parallel, vs. placebo *N* = 42	Fatigue Severity Scale (FSS)	200 mg QD 8?/10 weeks	No significant difference between treatment groups (similar significant improvements with both amantadine and placebo)
	Shaygannejad et al. (2012)	Multiple sclerosis with fatigue (mean duration 3 years)	RCT, Crossover, vs acetylsalicylic acid (500 mg) *N* = 52	Fatigue Severity Scale (FSS)	100 mg BID 10 weeks	No significant difference between treatment groups (similar significant improvements with both substances)
	Ledinek et al. (2013)	Multiple sclerosis with fatigue (mean duration 6.6 years)	RCT, Parallel, vs. Modafinil (200 mg) or acetyl-l-carnitine (2 g) *N* = 60	Modified Fatigue Impact Scale (MFIS)	200 mg QD 4 weeks	One-month treatment with amantadine improved fatigue in patients with relapsing–remitting MS as evaluated by MFIS. No or only a trend of improvement was seen in patients treated with modafinil or ALCAR, respectively
	Cohen and Fisher (1989)	Multiple sclerosis with fatigue	RCT, Crossover, vs placebo *N* = 29	Fatigue Assessment Inventory (FAI)	200 mg	No significant difference between treatment groups
	Rosenberg and Appenzeller (1988)	Multiple sclerosis with fatigue	RCT, Crossover, vs placebo *N* = 10	Fatigability Scale (0–4)	200 mg	Six subjects in the amantadine group and one subject in the placebo group reported improvement of fatigue symptoms
	Khazaei et al. (2019)	Multiple sclerosis with fatigue	RCT, Crossover, vs. Ondansetron *N* = 53	Fatigue Severity Scale (FSS) questionnaire	100 mg BID over 4 weeks	Both amantadine and ondansetron reduce fatigue in MS patients, but the efficacy of amantadine in reducing the MS-associated fatigue is greater than that of ondansetron
	Cohen et al. (2019)	Multiple sclerosis	RCT, double-blind, vs. Placebo *N* = 60 (amantadine * N* = 30)	Timed 25-Foot Walk (T25FW), Timed Up and Go (TUG), 2-min Walk Test, Multiple Sclerosis Walking Scale-12, fatigue, depression, and cognition, safety, and tolerability	274 mg QD over 4 weeks	ADS-5102 was well tolerated and improved walking speed
Tardive dyskinesia	Angus et al. (1997)	Tardive dyskinesia	RCT, crossover, vs. placebo *N* = 16	Abnormal Involuntary Movements Scale (AIMS)	300 mg over 18 weeks	15% Improvement on AIMS, * p* = 0.05)
	Decker et al. (1971)	Tardive dyskinesia	RCT *N* = 6	Crane quantitative scale	300 mg over 3 weeks	Improvement on Crane quantitative scale
	Pappa et al. (2010)	Tardive dyskinesia	RCT *N* = 22	Abnormal Involuntary Movements Scale (AIMS)	100 mg over 2 weeks (for 2 weeks followed by 4-day washout and 2 weeks of placebo)	21.8% improvement on AIMS (* p* = 0.000)
	Freudenreich and McEvoy (1995)	Tardive dyskinesia	Case report *N* = 1	Abnormal Involuntary Movements Scale (AIMS)	100 mg b.i.d	Decrease in AIMS score from 19 to 3
	Allen (1982)	Tardive dyskinesia	Case series *N* = 6	Abnormal Involuntary Movements Scale (AIMS)	100–300 mg/day	Palliative effect of combination amantadine/neuroleptic in tardive dyskinesia
Depression	Vale et al. (1971)	Chronic depressive syndrome	Randomized, double-blind, placebo-controlled 16–18/group	Zung Self-Rating Scale (SRS)	100–200 mg 4 weeks	67% of patients improved vs. 25% in placebo group Diagnostic criteria not used Response: score below median
	Stryjer et al. (2003)	Major depression (refractory)	Open-label, add-on *N* = 8	Hamilton Depression Rating Scale (HDRS), CGI = Clinical Global Impressions (CGI)	100–300 mg 4 weeks	50% of patients responded (≥ 50% reduction) Pre vs. post comparison
	Rogoz et al. (2007)	Major depression (refractory)	Open-label Comparison between imipramine and imipramine + amantadine *N* = 25/group	Hamilton Depression Rating Scale (HDRS)	100 mg 6 weeks	Potentiated effect of imipramine
	Ferszt et al. (1999)	Major depression, bipolar depression, Borna Disease virus infection	Open-label, add-on *N* = 30	Montgomery-Asberg Rating Scale (MADRS)	200–350 mg 8–12 weeks	63% of patients improved (≥ 40% reduction) Better response associated with Ag2 BDV antigen
	Dietrich et al. (2000)	Major depression, bipolar depression, dysthymia Borna Disease virus infection	Open-label, add-on *N* = 25	Hamilton Depression Rating Scale (HDRS)	100–300 mg 11 weeks (mean)	68% of patients improved (≥ 50% reduction or two steps on Operationalized Diagnostic Criteria System, OCPCRIT)
	Ziedonis and Kosten (1991)	Depression secondary to cocaine addiction	Randomized, double-blind, placebo, and active comparator (desipramine)-controlled *N* = 5–9/group	Beck Depression Inventory (BDI)	300 mg 12 weeks	Prevented increase in depression score, reduced cocaine craving, and consumption
	(Quarantini et al. (2006)	Depression induced by interferon-α	Open-label, add-on *N* = 6–8/group	Hospital Anxiety and Depression Scale (HADS)	200 mg 24 weeks	Prevented depression Exclusion criteria: history of depression
	Kronenberger et al. (2007)	Depression induced by interferon-α	Randomized, double-blind, placebo-controlled *N* = 131–136/group	Profile of Mood States (POMS)	200 mg 48 weeks	No effect on POMS depression factor Prevented depressive symptoms in a subset of patients

Yang et al. (2017) performed an extensive review of pharmacological treatments for fatigue in patients with MS and performed a meta-analysis on five studies comparing amantadine treatment with a placebo for fatigue in MS patients, indicating that amantadine might be the most effective drug for treating MS fatigue (Yang et al. 2017). Likewise, a recent meta-analysis including a total of 11 clinical trials with amantadine using both validated fatigue scales the patients’ subjective responses revealed improvement of MS-related fatigue with amantadine (Perez et al. 2020).

Consensus guidelines from the German Multiple Sclerosis Society (GMSS) conclude that amantadine produces moderate improvement in subjective fatigue, concentration, memory, and problem-solving compared with placebo based on strong evidence (Generali and Cada 2014).

Although several placebo-controlled trials have shown favorable results for subjective measures of fatigue associated with MS (see Table 5 for details), a Cochrane Database Review by Pucci et al. (2007) concluded that the efficacy and tolerability of amantadine in this population is poorly documented. They indicated the necessity of further improvement of knowledge on mechanisms of MS-related fatigue, determination of appropriate outcome measures, and new high-quality randomized clinical trials (Pucci et al. 2007). Also, studies demonstrating benefits in different subsets of patients (e.g., those with advanced illness) are still necessary.

### Olivopontocerebellar atrophy and Friedreich ataxia

#### Preclinical studies

In a coat-hanger test in lurcher mutant mice amantadine at 40 g/kg improved performance, a similar effect was observed after ketamine (Lalonde et al. 1993) indicating involvement of NMDA receptors.

#### Human studies

The initially promising case reports in patients with Friedreich’s ataxia, showing some improvements with amantadine in visual and auditory reaction times and movement times or on the Functional Ataxia Scoring Scale (FASS) (Botez et al. 1989; Peterson et al. 1988), failed to find their confirmation in later, larger double-blind placebo-controlled clinical trials using similar clinical tools (Botez et al. 1996; Filla et al. 1993). However, the study of Botez et al. (1996) shows significant improvements in visual and auditory reaction times and movement times with amantadine in a subset of patients with olivopontocerebellar atrophy (Botez et al. 1996). Further well-designed studies in this indication are warranted.

### ***Huntington***’***s disease***

#### Preclinical studies

We could not detect any studies of amantadine in animal models of Huntington’s disease.

#### Human studies

The first reports on putative beneficial effects of amantadine in Huntington’s disease chorea (Scotti and Spinnler 1971) were initially considered controversial (Weiner and Klawans 1972). Later randomized studies of Class II and Class I brought positive or mixed results, respectively (O'Suilleabhain and Dewey 2003; Verhagen Metman et al. 2002). Accordingly, the evidence-based guideline of American Academy of Neurology (AAN) concluded that blinded patient-reported outcomes in one Class I study, as well as one Class II study suggest that amantadine is likely effective in decreasing HD chorea (Armstrong and Miyasaki 2012). The above results found confirmation in further open-label and double-blind-placebo-controlled studies.

### Tardive dyskinesia

#### Preclinical studies

In one study, it was shown that amantadine co-treatment reduced the development of dopaminergic receptor hypersensitivity in the striatum after haloperidol administration as evidenced by analysis of stereotyped behavior and a D-2[^3^H]spiroperidol receptor binding assay (Allen et al. 1980). This suggests potential utility in the prevention of tardive dyskinesia but requires confirmation from further studies.

#### Human studies

Several clinical studies examined the effect of amantadine on patients with tardive dyskinesia (Allen 1982; Angus et al. 1997; Decker et al. 1971; Freudenreich and McEvoy 1995; Pappa et al. 2010). An 18-week, double-blind, crossover, randomized placebo-controlled trial reported amantadine (300 mg/day for 7 weeks) to be beneficial in tardive syndrome when co-administered with neuroleptics. In patients taking amantadine, tardive dyskinesia symptoms quantified using the Abnormal Involuntary Movements Scale (AIMS) were reduced by 15% (Angus et al. 1997). Pappa et al. (2010) administered amantadine (100 mg/day) in patients with tardive dyskinesia and stable psychiatric condition in a double-blind placebo-controlled study. The authors observed a highly significant reduction on total AIMS score by approximately 22%, as well as on individual AIMS items—including AIMS severity score—with amantadine but not with placebo (Pappa et al. 2010). In a small clinical trial involving 6 subjects, improvements were observed with amantadine on the quantitative scale by Crane (1968) and Decker et al. (1971). The remaining few studies comprise case reports showcasing beneficial effects of amantadine and pointing out the heterogenicity of the indication (Allen 1982; Freudenreich and McEvoy 1995).

Based on the results of the available clinical studies, an evidence-based guideline prepared by Guideline Development Subcommittee of the American Academy of Neurology (AAN) “Treatment of tardive syndromes” granted amantadine administered for a short term together with a neuroleptic the recommendation Level C, corresponding to “might be considered as tardive syndrome treatment” (Bhidayasiri et al. 2013, 2018). This guideline suggested that the evidence for amantadine’s efficacy exists only for the neuroleptics flupentixol decanoate, chlorpromazine, haloperidol, trifluoperazine, and thioridazine, as only these drugs were tested with amantadine.

### Neuroleptics-induced weight gain

#### Preclinical studies

In female rats, chronic administration amantadine for 3 weeks decreased body weight at 100 mg/kg (but not at 50) in naive rats and in rats treated with sulpiride. Hyperprolactinemia was not affected (Baptista et al. 1997). It should be stressed that 100 mg/kg is above therapeutically relevant dose and lack of effect on prolactin levels argue against the usefulness of amantadine for this indication.

#### Human studies

The most recent meta-analysis taking the results of 5 randomized clinical trials into consideration (for details see Table 5) demonstrated that administration of amantadine was associated with a 2.22 kg reduction in body weight (Zheng et al. 2017), which roughly remains in agreement with previous somewhat limited meta-analyses showing reductions of slightly less than 2 kg (Kishi and Iwata 2013; Praharaj and Sharma 2012). Results of the meta-analysis by Zheng and colleagues not only showed that amantadine was effectively reducing weight gain associated with antipsychotics therapy but is also safe and well tolerated in patients with schizophrenia. It should, however, be mentioned that metformin was associated with a weight reduction of 3.42 kg in a similar population (Zheng et al. 2017). A head-to-head study comparing the effects of amantadine and metformin could be envisaged.

### Pain

#### Preclinical studies

Subcutaneous treatment with amantadine with ED_50_ of 36.1 mg/kg decreased tactile nociception on the back of rats as measured using back skin twitches after von Frey filament stimulation (Chen et al. 2012). Since the effect was seen at therapeutically relevant dose, this finding is encouraging and warrants further preclinical investigations, and, if they are positive, they should be followed by clinical trial.

#### Human studies

The potential efficacy of NMDA receptor antagonists in neuropathic pain was suggested by several authors. Aiyer et al. (2018) performed an extensive review of publications from 58 randomized-controlled trials with clinically approved drugs with known NMDA antagonist or anti-glutamatergic properties. However, there is not much clinical evidence that amantadine may be useful for the treatment of pain of any type. Only two of three trials with amantadine considered showed analgesic properties in patients with neuropathic pain [for review, see (Aiyer et al. 2018)]. In a pilot study in subjects with diabetic neuropathy, amantadine reduced the pain of painful peripheral neuropathy [effect sustained for at least 1 week after the infusion (Amin and Sturrock 2003)]. Pud et al. demonstrated amantadine to statistically significantly relieve pain compared to placebo as well as to decrease “wind up”-like pain in patients with surgical neuropathic cancer pain (Pud et al. 1998). Medrik-Goldberg et al. (1999) compared spontaneous and evoked pain originating from the sciatic nerve pathway after treatment with lidocaine, amantadine, or placebo in a double-blind, randomized, controlled study. Lidocaine was significantly better than placebo or amantadine to relieve both kinds of pain (Medrik-Goldberg et al. 1999).

There are several further studies addressing effect of amantadine in pain of different origin. A double-blind, placebo-controlled trial of amantadine carried out in 100 patients with acute herpes zoster showed no difference between amantadine and placebo in the duration of pain that disappeared during the 28 days' observation period. However, pain duration exceeded 28 days in a significantly greater proportion of patients on placebo than of those on amantadine (Galbraith 1973). In the study of Chiba et al. (1992) in multiple sclerosis (MS) patients, over 70% of patients with pain showed a marked reduction in heavy and tingling pain in the face and the back mostly lasting for months (Chiba et al. 1992). On the other hand, preoperative infusion of amantadine (200 mg) failed to enhance postoperative analgesia in patients undergoing abdominal hysterectomy (Gottschalk et al. 2001). Similarly, perioperative oral administration of amantadine (100 mg BID) failed to prevent pain syndrome commonly associated with nerve injury following breast surgery with axillary lymph-node dissection (Eisenberg et al. 2007). Concerted preclinical and clinical efforts might elucidate the potential of amantadine in the treatment of different kinds of pain.

### Epilepsy

#### Preclinical studies

We could not localize any studies on amantadine in specific models of epilepsy. However, amantadine seems to have very weak anticonvulsive activity against convulsions in general. In mice, in maximal electroshock seizures, NMDA-induced seizures, and pentylenetetrazol seizures, ED50 of amantadine was, respectively, 184, 116, and > 100 mg/kg (Parsons et al. 1995).

#### Human studies

Shields et al. assessed the effect of add-on amantadine in 10 pediatric patients with refractory seizures over 12–16 weeks. Improvements in control of myoclonic or atypical absence seizures were observed in some of the patients (Shields et al. 1985). Likewise, in a more recent study in a cohort of pediatric patients, the efficacy of amantadine has been demonstrated in the treatment of refractory absence and myoclonic type seizures. To this end, the authors performed a retrospective review of medical records for patients with absence seizures who were treated with amantadine. The patients had been taking multiple antiepileptic drugs or had been implanted a stimulator of the vagus nerve. An ≥ 50% reduction in seizures was reported in more than 50% of patients after 3, 6, and 12 months of adjunctive treatment. A majority of responders had > 90% reduction in seizure frequency (Perry et al. 2012).

It can be concluded that amantadine may be useful as an add-on treatment in refractory atypical absence or myoclonic seizures. Further high-quality randomized-controlled trials are warranted.

### Sexual dysfunction

#### Preclinical studies

In male rats, amantadine (1.25–50 mg/kg) decreased latency for mounts and at higher doses decreased the number of mounts and intromission latency (Ferraz and Santos 1995). Lower doses also increased ejaculation latency and increased intromissions frequency. Some of these effects were attenuated by haloperidol and atropine.

Since there is only one study supporting this kind of activity of amantadine, it can be concluded that there is no sufficient preclinical evidence supporting amantadine utility in sexual dysfunction.

#### Human studies

Literature regarding putative effects of amantadine in sexual dysfunction is mostly confined to observations in patients undergoing antidepressant therapy experiencing anorgasmia, orgasmic delay, and hypoactive sexual desire as adverse effects of antidepressants [for review, see (Woodrum and Brown 1998)].

In a large retrospective study in patients treated with serotonin reuptake inhibitors, 57.9% (11/19) patients treated with amantadine (administered at the dose of 100 mg BID) reported no change or worsening, 15.8% (3/19) experienced “some improvement”, and 26.3 (5/19) were “much improved” (Keller Ashton et al. 1997). The improvements in amantadine-treated patients were comparable to those in patients receiving cyproheptadine but clearly less pronounced in that given yohimbine as “antidotes” (Keller Ashton et al. 1997).

There are also few case reports, suggesting that amantadine could be useful for the treatment of sexual dysfunction induced by serotonergic antidepressants (Balogh et al. 1992; Balon 1996; Masand et al. 1994; Shrivastava et al. 1995). However, there are also case reports evidencing the lack of effect of amantadine in such patients (Gitlin 1995). Likewise, in a randomized trial comparing effects of bupropion with those of amantadine over 4 weeks in patients with selective serotonin reuptake inhibitor (SSRI)-associated sexual dysfunction, the improvement in the bupropion group was significantly more pronounced than in the amantadine group (Zahiroddin et al. 2015).

It is worth mentioning that a small open-label drug study by Valevski et al. suggests that amantadine (100 mg daily over 6 weeks in 12 subjects) may also be effective for improving sexual function in male schizophrenic patients administered neuroleptics. Indeed, amantadine improved the patients' scores of desire, erection, and satisfaction from sexual performance. However, there was no change in ejaculatory function score (Valevski et al. 1998).

### Depression

#### Preclinical studies

Amantadine at 100 mg/kg attenuated reserpine-induced sedation in mice, an effect which was a few decades ago considered as indicative of antidepressive activity (Messiha 1988).

In rats, amantadine decreased reserpine-induced hypothermia (starting at 40 mg/kg) and attenuated increase in despair in forced swim test starting at 20 mg/kg (Moryl et al. 1993). In another study, amantadine at 20 mg/kg decreased immobility in forced swim test and enhanced effect of imipramine (Rogoz et al. 2004).

Combination of sigma1 or sigma2 agonists cutamesine (1-(3,4-dimethoxyphenethyl)-4-(3-phenylpropyl)piperazine dihydrochloride, SA4503) or siramesine (1′-[4-[1-(4-fluorophenyl)indol-3-yl]butyl]spiro[1*H*-2-benzofuran-3,4′-piperidine], respectively, with amantadine (10 mg/kg) produced stronger antidepressive-like effect in the forced swim test in rats than each treatment given alone at particular dose (Skuza and Rogoz 2002). In another study, the antidepressive effect of sigma1 ligands PB212 and PB190 was also enhanced by amantadine at 10 mg/kg in tail suspension test in mice or forced swim test in rats (Skuza et al. 2014).

In animals exposed to chronic unpredictable stress for 21 days, amantadine (25 mg/kg) administrated p.o. for 20 days from the 4th day to the 23^rd^ day increased sucrose consumption and attenuated spatial learning in Morris water maze (Yu et al. 2016). Amantadine also decreased impairment of hippocampal synaptic plasticity (LTP and depotentiation) and enhanced the expression of hippocampal NR2B and PSD-95 in stressed rats (ibid).

A different aspect related to amantadine on depression involves the postulated anti-Borna virus activity. In vitro, treatment of rabbit brain cells with amantadine inhibited human Borna virus (BDV-Hu-H1) at 0.2 µg/ml (Bode et al. 1997). The same study also involved a human experiment (see next section). This original finding was not replicated in further preclinical experiments and in clinical studies (see below). In monkey vero cells, in vitro infection with Borna virus was not affected by amantadine (up to 1 µg/ml) at concentrations 400-fold higher than active at influenza A (Hallensleben et al. 1997). Similarly, in the same study, amantadine at therapeutically relevant doses/concentrations failed to improve brain infections in BALB/c mice. Similar results were obtained in in vitro (cell lines) and in vivo (rats) preclinical experiments by Stitz and colleagues (Stitz et al. 1998). Another in vitro study also confirms a lack of activity up to 10 µM concentration (Cubitt and delaTorre 1997).

In summary, results from animal models suggest usefulness of amantadine in depression; however, it should be taken with caution, since translational predictability of animal models of depression is low.

#### Human studies

Potential of amantadine as treatment of depression was already recognized in the early 70 s of the twentieth century (Vale et al. 1971). Nevertheless, the studies conducted ever since do not allow drawing reliable conclusions due to their limitations, mainly small sample size and an open-label design [for review see (Raupp-Barcaro et al. 2018)].

In the earliest, randomized and placebo-controlled study of amantadine in depression, Vale et al. compared patients receiving antidepressant medications and amantadine (100–200 mg) for 4 weeks to patients receiving antidepressants and placebo, and observed an improvement with amantadine; the effect disappeared upon discontinuation of amantadine (Vale et al. 1971). Further two studies evaluated the efficacy of amantadine in treatment-resistant depression. Stryjer et al. evaluated amantadine (up to 300 mg/day for 4 weeks) as adjunct treatment in eight patients with treatment-resistant depression, but found no significant improvement in symptoms of depression (Stryjer et al. 2003). Rogoz et al. evaluated the effects of amantadine (150 mg BID) as an add-on drug to imipramine (100 mg/day) in treatment-resistant unipolar depression patients. The Hamilton Depression Rating Scale (HDRS) scores were not changed by imipramine alone, but they did decrease at 6 weeks with the combined administration of imipramine plus amantadine. Any contribution of a pharmacokinetic interaction to this effect was excluded (Rogoz et al. 2007). Repetitive treatment with imipramine and amantadine produced increase in D3 receptor binding in lymphocytes which correlated with symptoms improvement of depressive patients (Dziedzicka-Wasylewska et al. 2002).

In a very recent case study, amantadine (100 or 200 mg daily) improved depressive symptoms in four bipolar I disorder patients resistant to common antidepressants (Krzystanek and Palasz 2020).

Further studies evaluated the effects of amantadine in the treatment of secondary depression: in patients with hepatitis C receiving interferon treatment (Kronenberger et al. 2007; Quarantini et al. 2006), in cocaine addiction (Ziedonis and Kosten 1991), and in depression related to Borna disease virus infection (Dietrich et al. 2000; Ferszt et al. 1999). In most of these studies, amantadine given in a daily dose range 100–300 mg and combined with the patients’ usual medication improved depressive symptomatology.

The effects of amantadine observed in these few clinical studies are generally in alignment with the effects of this compound in different animal models of depression. Furthermore, some evidence suggests that amantadine may enhance the therapeutic effects of antidepressants. Given the preclinical evidence and the fact that the drug is being used in neurology, amantadine might be a promising choice for the treatment of depression. However, further controlled studies are still necessary to confirm this hypothesis.

### Anxiety

#### Preclinical studies

Amantadine at 50 and 75 mg/kg (but not 25 mg/kg) decreased anxiety in mice as shown in light–dark box and elevated plus maze tests (Walia et al. 2020). Interestingly, amantadine also decreased brain nitrate levels.

#### Human studies

To the best of our knowledge, no clinical studies specifically addressed the effects of amantadine in anxiety disorders. However, anxiety was assessed as one of the parameters in studies evaluating amantadine for the treatment of other indications. For example, improvements in anxiety scores accompanied amelioration of depressive scores in patients with treatment-resistant depression who were administered amantadine at the dose of 300 mg over 4 weeks (Stryjer et al. 2003). Anxiety was also reported to be one of the areas showing most consistent improvements in a retrospective review of the hospital charts of 12 subjects with brain injury who were treated with amantadine (Nickels et al. 1994). On the other hand, amantadine added to stable selective serotonin reuptake inhibitor (SSRI) regimen in patients with obsessive–compulsive disorder reduced total score and compulsivity subscale of Yale Brown Obsessive Compulsive Scale (Y-BOCS), but levels of anxiety and depression in those patients remained unaltered (Stryjer et al. 2014).

In conclusion, there is no sufficient evidence to justify the testing of amantadine in anxiety disorders.

### Other indications

Amantadine treatment also reduced B-cell lymphoma 2 protein (Bcl‑2) and increased the Bcl-2-associated X protein (Bax) and mRNA levels in hepatocellular carcinoma cell line increasing apoptosis (Lan et al. 2015). This could be a hint for utility in cancer treatment.

## Conclusions: perspectives on extension of therapeutic applications of amantadine

The first impression from re-evaluation of amantadine publications was that we should reappraise its MoA. If we consider amantadine’s affinity to different targets in vitro and expected concentration at these targets after therapeutic doses in humans (Fig. 2), AADC, GDNF, sigma-1 receptors, PDEs, and nicotinic receptors seem to be the most plausible primary therapeutic targets. NMDA receptors, 5-HT3 receptors, and potassium channels appear as further possible yet less feasible targets. Amantadine’s activity profile at these “new” receptors supports the expansion of its use to new indications reflected in published or ongoing (Table 5).

The authors believe that discussed clinical studies allow stating that beyond motor effects in Parkinson’s disease and ani-influenza effects, there are several encouraging indications that deserve further exploration in carefully designed and sufficiently powered randomized clinical trials. These include recovery after TBI, fatigue (e.g., in MS) related to neurostimulatory effect and tardive dyskinesia. It is noteworthy that amantadine is recommended in practice guidelines for disorders of consciousness, TBI recovery, MS fatigue, tardive dyskinesia, and Huntington’s disease (Armstrong and Miyasaki 2012; Bhidayasiri et al. 2018; Butterworth 2020a; Generali and Cada 2014; Giacino et al. 2018; Plantier and Luaute 2016).

Preclinical investigations suggest amantadine’s utility in several further indications; however, clinical confirmation of such activities is still lacking. Among these indications are recovery after spinal cord injury, MS, and cutaneous pain.

Query in the clinical study database http://www.clinicaltrials.gov reveals ongoing or planned studies in both established and potential indications. These studies sorted according to the number of occurrences could be summarized as follows: Cancer (15), Parkinson’s disease (15), L-DOPA-induced dyskinesia (7), autism (6), TBI (6), cocaine abuse (5), MS (5), diabetes (3), and ADHD, dental caries, obesity, and schizophrenia with two studies each; there are also isolated studies for several further indications.

The present reappraisal of pharmacological target profile and therapeutic potential of amantadine suggests that this drug is as timeless as a diamond and that further search for the extension of current indications seems to be justified.

## Abbreviations

5-HT: 5-Hydroxytrytamine (serotonin)
Ach: Acetylcholine
AMPA: (*S*)-α-Amino-3-hydroxy-5-methyl-4-isoxazolepropionic acid
ATP: Adenosine triphosphate
cAMP: Cyclic adenosine-monophosphate
DA: Dopamine
DTG: 1,3-Di-*o*-tolylguanidine
GABA: γ-Aminobutyric acid
GDNF: Glia-derived neurotrophic factor
Glycine_A_: Inhibitory glycine receptor
MAO: Monoamine oxidase
NA: Noradrenaline
NMDA: *N*-Methyl-d-aspartate
PDA: Phosphodiesterase
QNB: Quinuclidinyl benzilate
